# Modular organization of locomotor networks in people with severe spinal cord injury

**DOI:** 10.3389/fnins.2022.1041015

**Published:** 2022-12-07

**Authors:** Soo Yeon Sun, Simon F. Giszter, Susan J. Harkema, Claudia A. Angeli

**Affiliations:** ^1^Department of Physical Therapy, Alvernia University, Reading, PA, United States; ^2^Department of Neurobiology and Anatomy, College of Medicine, Drexel University, Philadelphia, PA, United States; ^3^School of Biomedical Engineering, Science and Health Systems, Drexel University, Philadelphia, PA, United States; ^4^Kentucky Spinal Cord Injury Research Center, University of Louisville, Louisville, KY, United States; ^5^Department of Neurological Surgery, University of Louisville, Louisville, KY, United States; ^6^Frazier Rehab Institute, University of Louisville Health, Louisville, KY, United States; ^7^Department of Bioengineering, University of Louisville, Louisville, KY, United States

**Keywords:** spinal cord injury, locomotion, synergy modules, locomotor networks, electromyography, spinal cord electrical stimulation

## Abstract

**Introduction:**

Previous studies support modular organization of locomotor circuitry contributing to the activation of muscles in a spatially and temporally organized manner during locomotion. Human spinal circuitry may reorganize after spinal cord injury; however, it is unclear if reorganization of spinal circuitry post-injury affects the modular organization. Here we characterize the modular synergy organization of locomotor muscle activity expressed during assisted stepping in subjects with complete and incomplete spinal cord injury (SCI) of varying chronicity, before any explicit training regimen. We also investigated whether the synergy characteristics changed in two subjects who achieved independent walking after training with spinal cord epidural stimulation.

**Methods:**

To capture synergy structures during stepping, individuals with SCI were stepped on a body-weight supported treadmill with manual facilitation, while electromyography (EMGs) were recorded from bilateral leg muscles. EMGs were analyzed using non-negative matrix factorization (NMF) and independent component analysis (ICA) to identify synergy patterns. Synergy patterns from the SCI subjects were compared across different clinical characteristics and to non-disabled subjects (NDs).

**Results:**

Results for both NMF and ICA indicated that the subjects with SCI were similar among themselves, but expressed a greater variability in the number of synergies for criterion variance capture compared to NDs, and weaker correlation to NDs. ICA yielded a greater number of muscle synergies than NMF. Further, the clinical characteristics of SCI subjects and chronicity did not predict any significant differences in the spatial synergy structures despite any neuroplastic changes. Further, post-training synergies did not become closer to ND synergies in two individuals.

**Discussion:**

These findings suggest fundamental differences between motor modules expressed in SCIs and NDs, as well as a striking level of spatial and temporal synergy stability in motor modules in the SCI population, absent the application of specific interventions.

## Introduction

Locomotion requires complex coordination of muscles in the body. It is a general consensus that the central nervous system (CNS) often controls the coordination by activating groups of muscles via networks of interneurons in the spinal cord, rather than controlling each of the muscles individually (Bernstein, 1967; Lundberg et al., 1987; Bizzi et al., 1995, 2000; McCrea and Rybak, 2008; Giszter, 2015). It has been suggested that descending signals combined with reflexes and local excitatory and inhibitory interactions of spinal circuitry dynamically select appropriate muscle synergies and behavior from the available repertoire (McCrea, 1992; Kargo and Giszter, 2000; Hart and Giszter, 2004; Cappellini et al., 2006; Krouchev et al., 2006; Ivanenko et al., 2007, 2013; Dominici et al., 2011; Fox et al., 2013; Yang et al., 2019; Sylos-Labini et al., 2020). Spinal interneurons that organize synergy patterns have been identified in frogs, mice and primates (Hart and Giszter, 2010; Takei and Seki, 2010; Levine et al., 2014; Takei et al., 2017), and intraspinal stimulation similarly revealed modular recruitment in animal models (Bizzi et al., 1991; Giszter et al., 1993; Mussa-Ivaldi et al., 1994; Tresch and Bizzi, 1999; Caggiano et al., 2016; Yaron et al., 2020). Further, modular organization of the spinal network is likely determined early in development and refined with experience (Dominici et al., 2011; Yang et al., 2019). Combined, these studies support modular organization of the spinal networks across species.

Muscle synergies are observed during locomotion in healthy individuals and following CNS injury. It is reported that people with complete and incomplete spinal cord injury (SCI) exhibited muscle synergies during treadmill walking (Ivanenko et al., 2013). The patterns of muscle weighting from individuals with motor-incomplete SCI resembled the normal pattern; however, the patterns from individuals with motor-complete SCI did not, indicating that the severity of the injury impacted the weighting in synergies (Ivanenko et al., 2013). In ambulatory individuals with incomplete SCI, fewer synergy modules and altered patterns of synergies were identified both in adults (Hayes et al., 2014; Perez-Nombela et al., 2017) and in pediatric populations (Fox et al., 2013), compared to non-disabled individuals. Danner et al. (2015) reported that step-like, synergistic electromyography (EMG) patterns in the leg muscles were induced by unpatterned epidural electrical stimulation in individuals with motor-complete SCI while supine, suggesting modular organization of the spinal circuitry can be expressed in this population even with unusual or altered feedback. In people post-stroke, fewer synergy modules were identified during walking as a result of merging of the modules observed in non-disabled individuals (Clark et al., 2010). These studies investigated the organization of modules when some supraspinal inputs are partially or completely lost after CNS injury. However, we know rather little about the synergies exhibited by the residual spinal locomotor circuitry (which is known to undergo anatomical and functional reorganization in both humans (Hiersemenzel et al., 2000; Schindler-Ivens and Shields, 2000; Beauparlant et al., 2013; Tseng and Shields, 2013) and animal models (de Leon et al., 1998, 1999; Rossignol et al., 1999, 2008; Pearson, 2000; Rossignol, 2006; Cote et al., 2012; Houle and Côté, 2013; Takeoka et al., 2014; Asboth et al., 2018), after motor-complete severe SCI, and with varying chronicity and neurological injury level. Interestingly, in rats, complete spinal transection occurring at different ages (neonate vs. adult) affords similar synergy patterns, that are nonetheless slightly different from intact adult synergy patterns, despite different experience and influences of descending pathways (Yang et al., 2019).

To better understand the modular organization of the locomotor network in the absence of voluntary stepping ability and its changes following the recovery of walking in humans, we here characterize the modular structure of the locomotor network expressed by the human spinal cord after SCI, and relate the modular structures to healthy patterns. We also explore if chronicity of the injury that allows extensive neuroplastic changes of the spinal circuitry affects the modular organization of the locomotor circuitry. Further, we examine whether clinical characteristics, such as the severity and neurological level of SCI, affect the modular organization. Finally, we asked whether and how the synergy characteristics changed when the subjects achieved walking after locomotor training with spinal cord epidural stimulation (scES, *n* = 2). Using non-negative matrix factorization (NMF) and independent component analysis (ICA), we investigate synergy profiles of leg muscle activity observed during stepping with manual facilitation and body weight support in people with SCI and non-disabled individuals. Our study provides information regarding the spatial and temporal profile of muscle synergies in a large sampling of people with SCI with varying clinical characteristics, prior to any systematic locomotor training, and demonstrates several consistent synergy structures despite these variations. Further, we show that the training with neuromodulation (scES) does not lead to the normalization of synergy patterns in the absence of neuromodulation, even though the individuals achieved independent walking with scES.

## Materials and methods

### Subjects

Eighty individuals were included in this study, consisting of two groups: 73 individuals with SCI (SCI group) and 7 non-disabled individuals (ND group). The SCI group (age 33.94 ± 12.92 years, YSI 4.30 ± 4.34 years, median YSI 3.0 years, IQR = 3.07 years) included 54 individuals with no motor function below the lesion (AIS A *n* = 34, AIS B *n* = 20), and 19 with an impaired motor function below the lesion (AIS C *n* = 13, AIS D *n* = 6). Table 1 and Supplementary Table 1 summarize the demographic and clinical characteristics of the subjects in the SCI group. All individuals in the SCI group were non-ambulatory, except 2 AIS D participants (D11 and D43) who ambulated with a walker. Removing these 2 ambulatory subjects from the data analysis did not affect any of the statistical findings presented here, and their modular patterns were similar to non-ambulatory subjects. We also present the data from two subjects (B23 and B30) who achieved independent walking in the presence of epidural stimulation following locomotor training with task-specific stimulation. The ND group (age 26.84 ± 3.57 years) had no diagnosis of neural injury and served as a control group to understand the characteristics of the modular organization of locomotor circuitry in the absence of spinal cord injury. The experimental protocol was approved by Institutional Review Board at the University of Louisville.

**TABLE 1:** Clinical characteristics of SCI subjects.

	Age (years)	Gender	YSI	NLI
AIS A (*N* = 34)	33.94 ± 11.72	26 male, 8 female	5.23 ± 5.59	Cervical = 16, Thoracic = 18
AIS B (*N* = 20)	32.30 ± 11.61	16 male, 4 female	3.26 ± 1.83	Cervical = 14, Thoracic = 5 Lumbar = 1
AIS C (*N* = 13)	34.46 ± 13.30	9 male, 4 female	4.13 ± 2.93	Cervical = 9, Thoracic = 4
AIS D (*N* = 6)	41.00 ± 18.84	4 male, female	2.88 ± 3.26	Cervical = 4, Thoracic = 2

YSI, years since injury, NLI, neurological level of injury.

### Data collection

Both ND and SCI groups stepped on the treadmill using partial body weight support. During stepping, surface EMG was recorded at 2,000 Hz using a custom-written LabView program (National Instruments, Austin, TX). EMGs from the soleus (SOL), medial gastrocnemius (MG), tibialis anterior (TA), medial hamstring (MH), vastus lateral (VL), rectus femoris (RF), and adductor magnus (AD) muscles were recorded bilaterally using bipolar surface electrodes with fixed inter-electrode distance (Motion Lab Systems, Baton Rouge, LA). Vertical ground reaction force (70 SCIs, 7 NDs) or foot switch data (3 SCIs) were also collected and used to identify the swing and stance phases of the step cycle. For post-training data (*n* = 2), the EMG data without epidural stimulation during treadmill walking with minimal assistance and body weight support were analyzed. Individuals in the ND group walked on the treadmill at varying speeds with body weight load (BWL), and EMGs during 1.07-1.34 meters/second (m/s) with 100% BWL were included in the analysis of the motor module. Individuals in the SCI group stepped on a body-weight supported treadmill with manual facilitation. Manual facilitation provided stepping-related sensory cues and helped optimize body kinematics. Step speed and percentage BWL were selected for each subject to produce the optimal kinematic pattern and muscle activation with manual facilitation. Foot switches with four sensors on each side were attached to the shoe insole to detect foot contact with the treadmill during stepping. Vertical ground reaction force data were acquired at a frequency of 100 Hz from the treadmill instrumented with pressure sensors (Zebris Medical GmbH, Germany).

### Data processing and analysis of motor module

Each channel of EMG signals was high-pass filtered at 40 Hz using a zero-lag fourth-order Butterworth filter, demeaned and rectified. The EMG signals were then low-pass filtered at 4 Hz using a zero lag, fourth-order Butterworth filter (Clark et al., 2010). Subsequently, the EMG from each muscle was normalized to its peak value from the gait cycle and time-interpolated over individual gait cycles on a time base with 100 points (Chvatal and Ting, 2012; Hayes et al., 2014) in order to allow comparisons between subjects. Motor modules were calculated based on the EMG traces of concatenated steps of ≥10 step cycles to capture step-to-step variability (Clark et al., 2010). In NDs, 10-19 steps were analyzed (13.29 ± 3.64 steps), and in SCIs, 10-34 steps were analyzed (18.03 ± 6.07 steps). After the EMG processing, motor modules during stepping were extracted separately for left and right leg muscles in each subject using three analysis methods: spatially-fixed non-negative matrix factorization (SF-NMF), temporally-fixed non-negative matrix factorization (TF-NMF), and independent component analysis (ICA). The number of modules required to account for ≥90% of the total variability in the EMG (VAF) was identified using each analysis (Clark et al., 2010).

#### Non-negative matrix factorization (NMF)

For both SF- and TF-NMF, the number of modules required to account for ≥90% of the total variability in the EMG (total VAF) was identified in each subject (Clark et al., 2010) using a custom-written MATLAB program. SF-NMF analysis was used to test the hypothesis that muscle synergies during stepping have fixed spatial patterns and varying temporal patterns (Safavynia and Ting, 2012). To identify spatially-fixed synergy patterns, the EMG data were structured in *m* x *t* matrices, where *m* is the number of muscles recorded from each leg, and *t* is the concatenated steps (100 points per cycle x number of step cycles). Then, the NMF algorithm was applied to the matrix, which generated spatially-fixed muscle weightings W (*m* x *n* matrix) and temporally-varying muscle recruitment pattern C (*n* × *t* matrix).

TF-NMF analysis yielded motor module descriptions used to explore the hypothesis that muscle synergies during stepping have fixed temporal patterns and varying spatial patterns (Safavynia and Ting, 2012). To identify temporally-fixed synergy patterns, the EMG data were structured in *s* x *r* matrices, where *s* is the number of time points, and *r* is the number of muscles x step cycles. When the NMF algorithm was applied to the matrix, temporally-fixed muscle recruitment pattern C (s × *n* matrix) and spatially-varying muscle weightings W (*n* x *r* matrix) were generated (Ivanenko et al., 2003; Safavynia and Ting, 2012).

#### Independent component analysis (ICA)

Independent component analysis was used to extract mutually independent synergy components from EMG data (Bell and Sejnowski, 1995; Hart and Giszter, 2004; Dominici et al., 2011; Yang et al., 2019). The value of ICA is to detect weakly excited synergies using information rather than variance (Yang et al., 2019). As many components as input muscles are extracted on the basis of minimal information of components. However, ICA is very sensitive to information content and the original sampling and filtering of EMG. It has been very effective with intramuscular recordings (Kargo and Nitz, 2003; Hart and Giszter, 2004; Krouchev et al., 2006; Yang et al., 2019) though often also effective in surface EMG (Cappellini et al., 2006). Here, we tested ICA to explore if it added valuable information to the variance-based analyses. ICA always provides as many modules as muscles, which can then be examined for variance contributions. ICA was performed using the code from Giszter lab (adapted from Makeig’s EEGlab; (McCrea, 1992; Makeig et al., 1997; Cappellini et al., 2006; Krouchev et al., 2006; Ivanenko et al., 2013; Yang et al., 2019). To be consistent with the synergy selection criterion in NMF analyses, the number of motor modules was identified based on the cumulative VAF ≥ 90%. In addition, to find a breakpoint or an “elbow” in the ICA VAF, a piecewise linear regression was performed using a piecewise regression function in OriginPro (Version 2021, OriginLab Corporation, Northampton, MA, USA).

#### Grouping of motor modules

The temporal and spatial characteristics of motor modules in NDs and SCIs were examined by grouping motor modules from each participant. Modules were grouped based on the muscle weighting and recruitment patterns with a high correlation coefficient (Pearson *r* ≥ 0.7). We did not set the ND patterns as a reference due to the possibility that the common synergy patterns in SCIs differ from those of NDs. Modules identified by SF-NMF were grouped based on the correlation of spatial patterns, whereas modules from TF-NMF were grouped based on the correlation of temporal patterns.

#### Analysis of post-training data

In the individuals who achieved voluntary walking post-training, we further compared their pre-training and post-training (no stimulation) data. Six out of 7 muscles (RF, VL, MH, TA, MG, and SOL) were in common between pre-training data and post-training data. Therefore, 7 AIS B participants were randomly selected to define muscle synergies for non-ambulatory SCIs using NMF analyses (SF and TF-NMF). Further, muscle synergies from 6 muscles were defined in all NDs (*n* = 7) using NMF analyses. We quantified the similarity of the sets of synergies in pre- and post-training in 2 subjects with those of NDs and a cohort of AIS B SCI individuals in free order, by calculating an inner product column similarity measure using the matcorr and matperm function in MATLAB (from EEGlab; (Makeig et al., 1997; Godlove et al., 2016; Yang et al., 2019). Ratios of the correlation for each pre- and post-training module were calculated by dividing its correlation to ND group mean synergy by its correlation to SCI group mean synergy. The ratio of greater than 1 indicates that the module exhibits greater similarity to the NDs than to the SCIs, and less than 1 indicates the module exhibits greater similarity to the SCIs than to the NDs.

### Statistical analysis

For descriptive and inferential statistics, the clinical characteristics, such as NLI and chronicity of injury (YSI), were further categorized to determine the effects of clinical characteristics on the number and profile of motor modules. NLI was categorized into upper cervical (C1-C4), lower cervical (C5-C8), upper thoracic (T1-T6), and lower thoracic (T7-T12) lesions. Categorization of thoracic lesions was based on the potential impact of the autonomic dysfunction present in subjects with T6 injury and above on the modular organization and spinal plasticity. A subject with a lumbar lesion (*n* = 1) was not categorized. YSI was categorized into 5 subgroups: ≤ 1 year, 1-2 years, 2-5 years, 5-10 years, and ≥ 10 years. to compare the effects of chronicity of the injury on motor modules. Group differences in the number of modules across synergy extraction methods in the SCIs were analyzed using a 3-way ANOVA or MANOVA (SPSS, Version 26.0. IBM Corp., Armonk, NY). A repeated-measures ANOVA was used to compare the number of modules identified by the 3 analyses. Wilcoxon signed-rank test and paired *t*-test were used to compare the number of modules found bilaterally in NDs and SCIs, respectively. The significance level was set at 0.05, and *post hoc* comparisons were performed using Bonferroni correction. The comparisons of the muscle weighting and recruitment patterns among individuals were conducted using a Pearson correlation. Data are presented as mean ± standard deviation (SD).

## Results

We analyzed data recorded from non-disabled individuals (NDs, *N* = 7) and SCI subjects (SCIs, *N* = 73; clinical characteristics in Table 1 and Supplementary Figure 1). Of those SCIs, 71 were non-ambulatory, while 2 American Spinal Injury Association Impairment Scale (AIS) D subjects were ambulatory with a walker. None were subject to any in-house locomotor training prior to the testing. Consistent with previous findings, subjects with motor-complete and incomplete SCI showed patterns of rhythmic leg muscle activity during stepping with manual facilitation (Dietz et al., 1995; Dobkin et al., 1995), allowing extraction of muscle synergies (Supplementary Figure 1). Fifty-one SCI subjects demonstrated irregular activity or inactivity in at least one muscle during stepping. Note that in both NMF and ICA these “noise patterns” were segregated by the synergy separation algorithms and account for the fractions of ungrouped synergies excluded. They are thus not a confound in our data. In addition, individuals with SCI showed greater step-to-step variability in vertical ground reaction force during stepping. The standard deviation of vertical ground reaction force was 5.21 ± 3.58% BWL on average (range 1.74-30.18%) in SCIs, whereas in NDs, the standard deviation of vertical ground reaction force was 3.35 ± 0.83% BWL on average (range 2.01-4.85%).

### Characteristics of stepping in individuals with SCI

We first examined if there was a specific set of step parameters that produced optimal stepping patterns with manual facilitation in individuals with SCI, and if the parameters differed depending on the clinical characteristics. The step speed and% body weight load (% BWL) were related to categorizations by AIS, years since injury (YSI), and neurological level of injury (NLI), and are shown in Supplementary Figure 2. A three-way MANOVA using AIS, YSI, and NLI indicated a significant interaction among the three factors on speed and% BWL, *F*(4, 74) = *3.71 p* = *0.008*. However, neither of the clinical characteristics individually had a significant effect on the step speed and% BWL (AIS *p* = *0.08*; YSI *p* = *0.08*; NLI *p* = *0.39*). *Post hoc* analysis using Kruskal-Wallis tests also indicated that the AIS, YSI, and NLI had no significant effects on speed or % BWL in our subjects (all *p* > *0.1*).

### Number of motor modules from SF-NMF, TF-NMF, and ICA

**FIGURE 1:**
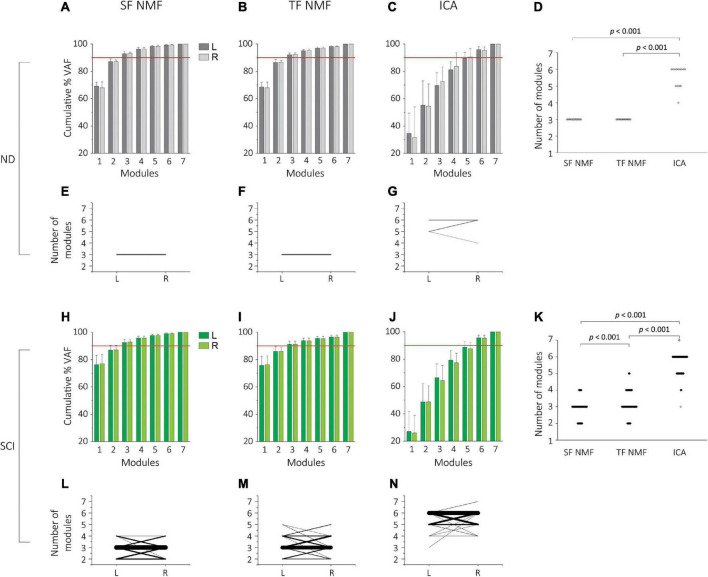
Cumulative percentage variability accounted for (%VAF) and the number of modules extracted by SF-NMF, TF-NMF, and ICA in NDs and SCIs. **(A–G)** indicates VAF and the number of modules in NDs (*N* = 7, 14 legs), and **(H,N)** indicates SCI data (*N* = 73, 146 legs). **(A–C)** %VAF (group mean + SD) for left (L) and right (R) legs from SF-NMF **(A)**, TF-NMF **(B)**, and ICA **(C)** in NDs. The horizontal red line indicates 90%. ICA required a greater number of modules to explain 90% VAF in EMGs. **(D)** Comparison of the number of modules explaining ≥90% VAF in the EMG all three analyses in NDs (left and right pooled). Each dot indicates an individual data point. SF- and TF-NMF identified 3 modules in all NDs, whereas ICA identified 4 modules in 1 leg (1 individual), 5 modules in 4 legs (4 individuals), 6 modules in 9 legs (6 individuals). ICA yielded a significantly greater number of modules compared to SF and TF-NMF to explain ≥90% VAF in NDs (*p* < 0.001). **(E-G)** The number of left and right modules in NDs, identified by SF-NMF **(E)**, TF-NMF **(F)**, and ICA **(G)**. Each line represents left-right pair for each subject. Thicker lines represent a greater prevalence of the pair. All subjects had 3 modules bilaterally from SF- and TF-NMF, whereas only 3 ND subjects had the same number of modules (Bizzi et al., 2000) bilaterally in ICA. The number of modules between left and right legs was not statistically different. **(H-J)**%VAF (group mean + SD) for left (L) and right (R) legs from SF-NMF **(H)**, TF-NMF **(I)**, and ICA **(J)** in SCIs. Similar to the NDs, ICA required a greater number of modules to explain 90% VAF in SCIs. **(K)** Comparison of the number of modules explaining ≥ 90% VAF in the EMG all three analyses in SCIs. SF-NMF yielded 2 modules in 32 legs (25 individuals), 3 modules in 97 legs (64 individuals), and 4 modules in 17 legs (16 individuals). TF-NMF yielded 2 modules in 19 legs (15 individuals), 3 modules in 85 legs (56 individuals), 4 modules in 35 legs (29 individuals), and 5 modules in 7 legs (7 individuals). ICA yielded 3 modules in 1 leg (1 individual), 4 modules in 7 legs (6 individuals), 5 modules in 44 legs (36 individuals), 6 modules in 92 legs (61 individuals) and 7 modules in 2 legs (2 individuals). ICA resulted in a significantly greater number of modules than SF- and TF-NMF in SCIs, and TF-NMF resulted in a greater number of modules than SF-NMF (*p* < 0.001). **(L-N)** The number of left and right modules in SCIs, identified by SF-NMF **(L)**, TF-NMF **(M)**, and ICA **(N)**. Each line represents left-right pair for each subject, and thicker lines represent a greater frequency of the pair. The number of modules between left and right legs was not statistically different.

We used spatially-fixed NMF (SF-NMF), temporally-fixed NMF (TF-NMF), and ICA to extract synergy patterns. These yielded varying numbers of motor modules to reliably reconstruct ≥90% variances accounted for (VAF) in the stepping EMG patterns in the NDs and SCIs. In all ND subjects, the SF-NMF and TF-NMF resulted in 3 modules to explain ≥90% VAF in each leg (Figures 1A,B), whereas the ICA identified 4-6 modules to explain ≥90% VAF (Figure 1C). Four modules were identified in 1 leg, 5 modules in 4 legs (4 individuals), and 6 modules in 9 legs (6 individuals). A repeated-measures ANOVA indicated that different modular analyses resulted in a significant difference in the number of modules in NDs detected, *F*(2, 26) = *221, p* < *0.001* (Figure 1D). *Post hoc* comparison indicated that the number of modules from ICA was significantly greater than those from SF and TF-NMF analyses (*Bonferroni p* < *0.001*; Figure 1D).

The number of motor modules was also compared between left and right legs to examine potential laterality or “sidedness” revealed by the analysis of motor modules. While all 7 NDs had the same number of modules bilaterally from both SF-NMF and TF-NMF (Figures 1E,F, respectively), in ICA, only 43% had the same number of modules bilaterally (Figure 1G). However, the number of motor modules extracted from ICA was not statistically different bilaterally (Wilcoxon signed rank test, *Z* = *0.80, p* = *0.38*).

Subjects in the SCI group exhibited a wider range of the number of motor modules, compared to NDs, needed to explain ≥90% VAF in all analyses. SF-NMF analysis led to 2-4 modules (mean 2.90 ± 0.57, Figure 1H), and TF-NMF led to 2-5 modules (mean 3.21 ± 0.73; Figure 1I). SF-NMF identified 2 modules in 34% of SCIs, 3 modules in 88%, and 4 modules in 22%. In TF-NMF, 2 modules were found 21% of SCIs, 3 modules in 77%, 4 modules in 40%, and 5 modules in 10%. Similar to the NDs, the ICA in the SCIs indicated a greater number of modules, resulting in 3-7 modules (mean 5.59 ± 0.64; Figure 1J). Three modules were identified in 1% of SCIs, 4 modules in 8%, 5 modules in 49%, 6 modules in 84%, and 7 modules in 3%. A repeated-measures ANOVA again indicated that different modular analyses resulted in a significantly different number of modules in SCIs, *F*(2,286) = *1382.40, p* < *0.001* (Figure 1K). *Post hoc* comparison indicated that the number of modules from all 3 analyses was significantly different from each other (*Bonferroni p* < *0.001*; Figure 1K). Results from NDs and SCIs together indicated that the ICA resulted in a greater number of modules compared to SF- and TF-NMF. This was consistent with ICA capturing weaker activated modules in the information-based analyses and likely richer drive structure, but the reduced number of recorded muscles and the surface EMG signal properties made these highly granular ICA results less informative than in other studies. Thus, subsequent analyses described here are focused on NMF.

The cumulative percentage VAF (% VAF) from SF- and TF-NMF demonstrated a clear “elbow” or breakpoint to determine the number of motor modules both in the NDs (Figures 1A,B) and SCIs (Figures 1H,I); however, cumulative% VAF found from ICA did not show the same characteristics (Figures 1C,J), and breakpoints were relatively poorly defined. Fuller and detailed comparison of NMF and ICA method for these data are in Supplementary material (Supplementary Results).

While some bilateral asymmetry is usually assumed *a priori* in SCIs, we directly compared the number of modules for the left and right leg in SCIs (Figures 1L–N). Forty-one SCI (56%) in SF-NMF, 37 SCIs (51%) in TF-NMF, and 39 SCIs (53%) in ICA had the same number of modules bilaterally. Paired *t*-tests indicated no significant laterality and associated bilateral asymmetry in the number of modules in the SCIs (*df* = *72, t* = *0.17, p* = *0.87* for SF-NMF*; df* = *72, t* = *−0.3, p* = *0.77* for TF-NMF*; df* = *72, t* = *−0.29, p* = *0.77* for ICA).

While the results of the left-right comparison indicated no sidedness, significant bilateral asymmetry may be present in the NDs and SCIs, absent systematic laterality, if the population sidedness was randomly sorted. Thus, we sorted the legs in each subject by fewer vs. more modules, i.e., regardless of the left or right, and tested if bilateral asymmetry was present and statistically significant in NDs and SCIs. When the number of modules between legs that had fewer vs. more modules was compared, there was no asymmetry in NDs (*Z* = *1.80, p* = *0.13*). However, in the SCI group, results indeed indicated significant bilateral asymmetry for SF-NMF (paired *t*-test, *t* = *−7.31, d.f.* = *72, p* < *0.001*) and for TF-NMF (*t* = *−7.71, d.f.* = *72, p* < *0.001*). Similarly, in ICA, there was also a significant bilateral asymmetry in SCIs (*t* = *−6.95, d.f.* = *72, p* < *0.001*). Thus, SCI resulted in significant bilateral asymmetry in the spinal circuitry that led to significant differences in motor modules between sides during stepping, but no laterality was observable. Lack of any systematic laterality allowed pooling of left and right legs into a single group for several of the subsequent analyses examining muscle weightings (spatial) and recruitment (temporal) patterns of motor modules.

### Clinical characteristics do not determine the number of motor modules

We examined if the clinical characteristics, including AIS, NLI, and YSI influenced the number of motor modules in individuals with SCI. Supplementary Figure 4 represents the percentage distribution of each number of modules across AIS (Supplementary Figure 4A), NLI (upper and lower cervical or thoracic level; Supplementary Figure 4B), and YSI (≤ 1 year, 1-2 years, 2-5 years, 5-10 years, ≥ 10 years; Supplementary Figure 4C), respectively. Three-way MANOVAs tested the effects of clinical characteristics on the left and right number of modules (separately) assessed from each of the analyses. Results for SF-NMF indicated no significant interaction among the three clinical characteristics, *F*(4, 74) = *0.86, p* = *0.50*, nor were main effects significant, *F*(6, 74) = *1.44, p* = *0.21; F*(8, 74) = *0.95, p* = *0.49; F*(8, 74) = *1.34, p* = *0.23*, respectively. Results for TF-NMF and ICA were similar, indicating that there were no significant interactions and no main effects of the clinical characteristics on the number of left and right modules, similar to the finding of clinical characteristics being unrelated to functional measures of %BWL and walking speed.

### Characteristics of motor modules identified by SF-NMF in NDs and SCIs

Spatially-fixed non-negative matrix factorization identified 3 modules in NDs and 2-4 modules in SCIs. In NDs, all the synergy patterns had a within-group correlation coefficient of ≥0.7 when cross-correlated and grouped; therefore, no patterns were excluded. However, in SCIs, 88.2-92.2% of the spatial pattern showed a correlation coefficient of ≥0.7. Further details on the excluded patterns are found in Supplementary material (Supplementary Results).

**FIGURE 2:**
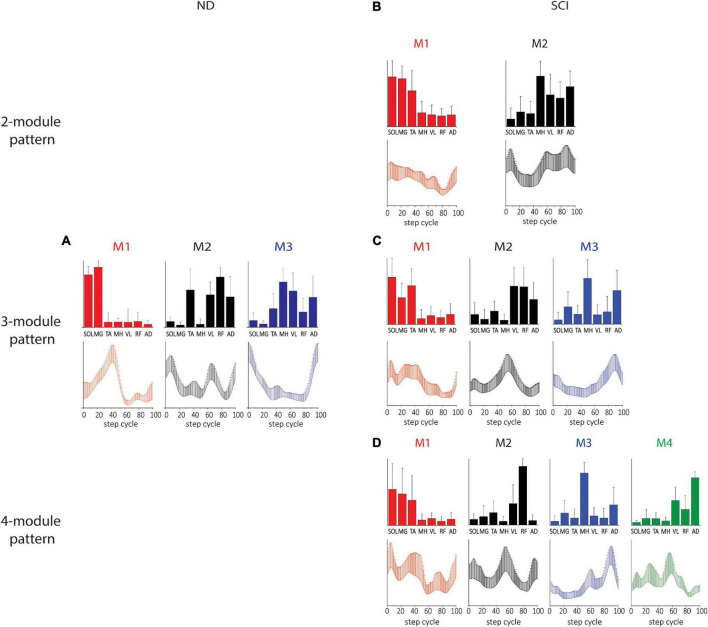
Spatial and temporal pattern identified by SF-NMF in NDs and SCIs. **(A)** ND 3-module pattern. **(B–D)** SCI 2-, 3-, and 4-module patterns. The correlations between the ND and SCI 3-module patterns were weak to moderate. Pearson r for M1 was 0.60 for the spatial patterns and 0.65 for the temporal patterns. For M2, Pearson r was 0.78 for spatial patterns and 0.03 for temporal patterns. For M3, Pearson r was 0.61 and 0.35 for spatial and temporal patterns, respectively. Bar graphs for spatial patterns indicate the group mean + SD. Temporal patterns are also indicated by group mean + SD. SOL, soleus; MG, medial gastrocnemius; TA, tibialis anterior; MH, medial hamstring; VL, vastus lateralis; RF, rectus femoris; AD, adductor.

The spatial and temporal patterns obtained from SF-NMF are presented in Figure 2. Spatial (synergy muscle weight) matching of modules extracted by SF-NMF resulted in a low variance in spatial patterns but greater variance in temporal patterns. Even though most muscles were active in more than 1 module, they were predominantly active in one module, as indicated by their weightings in the spatial pattern. We classified SCI subjects’ leg modularity by the number of modules capturing 90% VAF and examined the resulting groups of synergies in 2-module, 3-module, and 4-module patterns. In NDs, only 3-module patterns were identified.

In the 3-module pattern for NDs, Module 1 (M1) was characterized by SOL and MG activity during the mid-late stance, Module 2 (M2) was characterized by TA, VL, RF, and AD activity during the early stance and early and late swing, and Module 3 (M3) was characterized by MH, VL, and AD activity during the swing-to-stance transition (Figure 2A). In SCIs, all spatial and temporal profiles of motor modules looked somewhat different from those of NDs. In SCI 2-module patterns, each module was predominantly tied to stance or swing, with M1 active during the stance comprising SOL, MG, and TA activity and M2 active during the early stance and swing phases comprising MH, VL, RF, and AD activity (Figure 2B). In SCI 3-module patterns, while the spatial and temporal patterns of M1 appeared similar to the 2-module M1, we found that M2 and M3 diverged from the spatial pattern of the 2-module M2. During late stance in M2 in this case, VL and RF were active (M2), while MH and AD were active in M3 in late swing (M3; Figure 2C). In the SCI 4-module pattern (Figure 2D), the spatial and temporal patterns of M1, M2, and M3 appeared similar to those in the SCI 3-module patterns, and an M4 pattern appeared to have diverged from the 3-module M2. The correlation between the 3-module patterns of the NDs and SCIs was moderate (see Figure 2 legend for details). The differences in the temporal and spatial patterns between NDs and SCIs here indicate clear differences of synergy, e.g., coactivation of antagonistic ankle muscles during the stance phase occurs in the SCIs.

### Characteristics of motor modules identified by TF-NMF in NDs and SCIs

**FIGURE 3:**
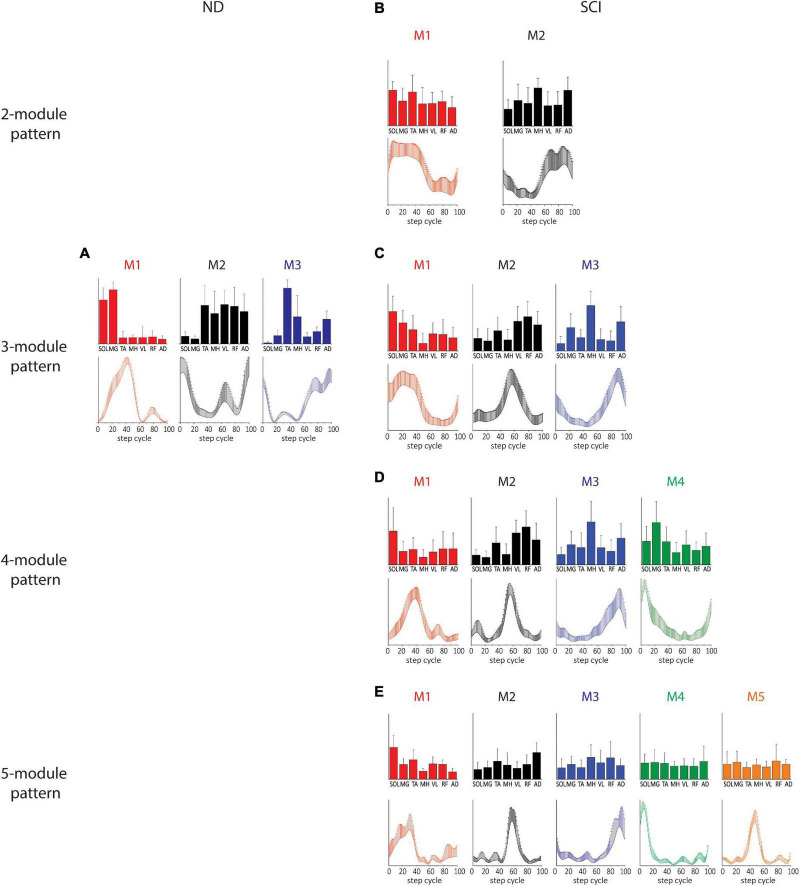
Spatial and temporal patterns identified by TF-NMF in NDs and SCIs. **(A)** ND 3-module pattern. **(B–E)** SCI 2-, 3-, 4-, and 5-module patterns. The correlations between the ND and SCI ND 3-module patterns were: for M1, temporal pattern *r* = 0.79 and spatial pattern *r* = 0.82; for M2, temporal pattern *r* = –0.30 and spatial pattern *r* = 0.75; for M3, temporal pattern *r* = 0.83 and spatial pattern *r* = 0.25.

Temporal matching in the modules extracted by TF-NMF resulted in a lower variance in temporal patterns and a greater variance in spatial patterns. Only 3 modules were identified by TF-NMF in all NDs. M1 was active during the mid-late stance with SOL and MG activity, M2 was active during the early swing and swing-to-stance transition with TA, MH, VL, RF, and AD activity, and M3 was active during the early stance and early and late swing phases with TA activity (Figure 3A).

Modules identified by TF-NMF in SCIs further revealed differences in the modular organization, also identifying more (Lundberg et al., 1987; Bizzi et al., 1995, 2000; McCrea and Rybak, 2008) module patterns (Figures 3B–D). In correlating temporal patterns, 88.6-91.4% of temporal patterns showed a correlation coefficient of ≥0.7 (further details in Supplementary material (Supplementary Results). In 2-module patterns, while temporal patterns indicated a synergy during stance (M1) and swing phase (M2), the associated spatial patterns did not show any clear distinction in muscle activation for each (Figure 3B). In 3-module patterns, M1 was characterized by SOL and MG activity during the early-mid stance, M2 by VL, RF, and AD activity during the stance-to-swing transition, and M3 by MH and AD activity during late swing (Figure 3C). The correlation coefficients between the ND and SCI 3-module patterns were only weak to moderate. In 4-module patterns, M1 and M4 appeared to have diverged from the M1 in 2- and 3-module temporal patterns (Figure 3D). In 5-module patterns, while temporal patterns indicated peaks in different phases of stepping, spatial patterns again lacked clear distinctions in muscle activity (Figure 3E). Interestingly, in 4- and 5-module patterns, the M1 temporal patterns resembled the M1 of NDs, although the spatial pattern from TF-NMF did not show any similarity.

### Correlation of motor modules identified by SF- and TF-NMF

In order to investigate if different strategies of identifying synergy patterns lead to different patterns of motor modules, we examined (for both SF- and TF-NMF analyses) the correlations between the within-method average temporal and spatial patterns of motor modules in NDs. M1 and M2 identified by both the two analyses in ND demonstrated a high correlation among subjects. For M1 of SF-NMF and TF-NMF, Pearson r for both temporal and spatial patterns were 1.00, and for M2, Pearson r for the spatial patterns was 0.78, and the temporal patterns 0.80. However, for M3, the correlation was weak; the correlation coefficient for the spatial patterns was 0.16, and the temporal patterns 0.42. These results indicate that while M1 and M2 in NDs were commonly identified as synergy patterns during stepping regardless of whether the spatial or temporal pattern was fixed, the patterns of M3 were more specific to the analysis method in ND.

We next compared correlations between the SF-NMF and TF-NMF methods. In SCIs, temporal and spatial patterns derived from SF-NMF and TF-NMF were compared within each of the 2-, 3-, and 4-module groups. In the SCI 2-module patterns, both M1 and M2 were highly correlated between SF- and TF-NMF methods. The correlation for M1 spatial patterns was 0.79, and temporal patterns 0.88. The correlation for M2 spatial patterns was 0.75, and temporal patterns 0.92. The SCI 3-module patterns also showed a high correlation between SF and TF-NMF. In M1, the correlation for spatial patterns was 0.97, and the temporal pattern was 1.00. The correlations for M2 spatial and temporal patterns were 0.97 and 0.96, respectively. Further, the correlation for M3 spatial patterns was 0.86, and temporal patterns 0.95. In SCI 4-module patterns, the correlation for M1 spatial patterns was 0.63, and temporal patterns 0.55. The correlation for M2 spatial patterns was 0.78, and temporal patterns 0.75. For M3, the correlation for the spatial patterns was 0.98, and the temporal pattern was 0.96. However, for M4, the correlation for the spatial and temporal patterns was −0.24 and −0.18, respectively. These results in the SCI group suggest that the modules identified in 2- and 3-module patterns were reliably identified regardless of whether the spatial or temporal pattern was fixed. However, the M4 pattern identified in the richer, 4-module pattern in SCI was more specific to the analysis method. The data as a whole support repeatable synergies and activation patterns across individuals in the untreated SCI population.

### Characteristics of motor modules across clinical characteristics using three modules

While the number and shape of synergy modules extracted based on ≥90% VAF criterion provided valuable information on the characteristics of the modular organization of stepping circuitry in NDs and SCIs, the variations in module number resulting made it difficult to compare the motor modules across all SCI subjects. Thus, we proceeded to run a version of the NMF analysis using a 3-modules analysis on all subjects. This allowed us to further examine the effects of NLI, AIS, and chronicity of injury on the characteristics of motor modules.

**FIGURE 4:**
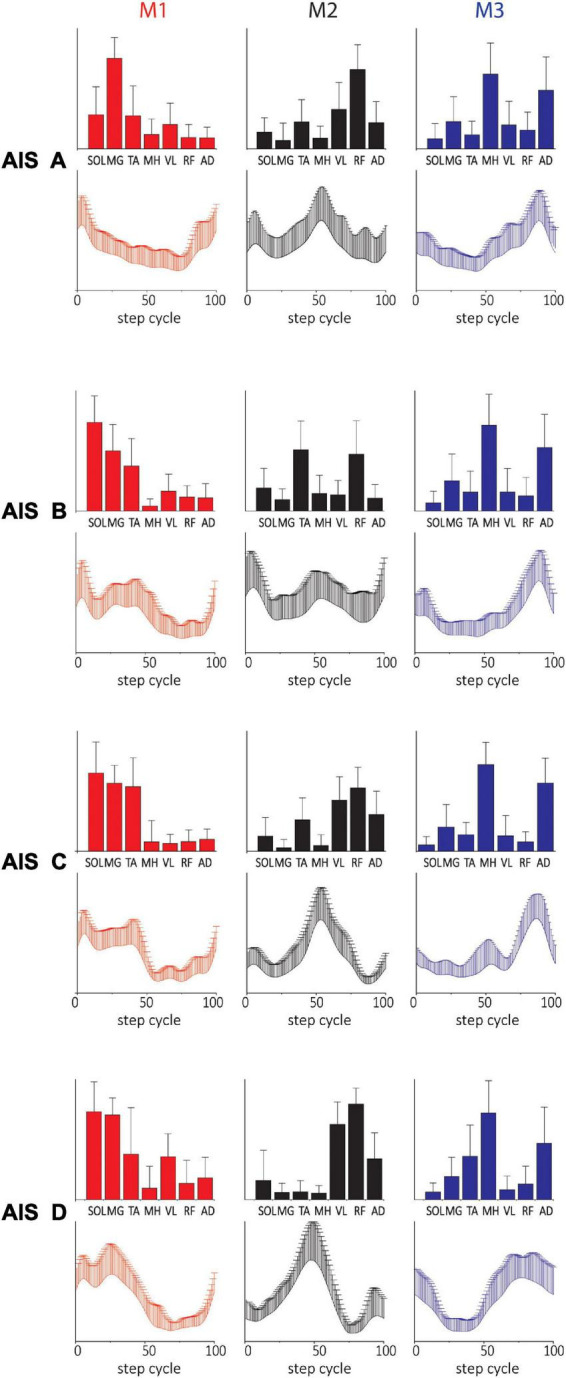
Three-(fixed) modules extracted from SF-NMF sorted by AIS. Each panel shows spatially-fixed synergy patterns during stepping categorized by AIS grade (group mean + SD). The patterns were generally well correlated across AIS categories. See Table 2 for details on correlation.

The spatial and temporal patterns identified by the added 3-module SF- and TF-NMF analyses were mostly similar across AIS (SF-NMF modules in Figure 4, TF-NMF modules in Supplementary Figure 6). The majority of the spatial and temporal profiles of each AIS subgroup also demonstrated moderate to strong correlation with each other (Supplementary Table 2). Results also indicated that the spatial and temporal profiles of the subgroups showed a weak or (for temporal) even negative correlation to ND synergies, except in spatial patterns of M1 and M2 from SF-NMF (cf. Figures 2A, 3A, 4 and Table 2) and temporal pattern of M3 from TF-NMF (cf. Figures 2A, 3A and Supplementary Figure 6, Supplementary Table 2). Further, the correlations of the patterns between NDs and AIS C and D subjects were similar to the correlations between NDs and ASI A and B subjects, indicating functional supraspinal control of leg muscles does not lead to stepping synergy patterns that better resemble those of NDs.

**TABLE 2:** Correlation of SF-NMF 3-module patterns among ND and AIS groups.

	Correlation of spatial patterns					Correlation of temporal patterns			
			
M1	ND	AIS A	AIS B	AIS C	M1	ND	AIS A	AIS B	AIS C
AIS A	**0.81** *				AIS A	−0.28*			
AIS B	**0.85** *	0.61			AIS B	0.61*	0.49*		
AIS C	**0.77** *	0.68	**0.94** *		AIS C	0.50*	0.64*	**0.93** *	
AIS D	**0.91** *	0.78*	**0.94** *	**0.87** *	AIS D	0.55*	0.54*	**0.92** *	**0.97** *

**M2**					**M2**				

AIS A	**0.82** *				AIS A	0.13			
AIS B	0.69	0.61			AIS B	**0.81** *	0.58*		
AIS C	**0.91** *	0.92*	0.49		AIS C	0.10	**0.97** *	0.50*	
AIS D	0.68	0.90*	0.24	**0.91** *	AIS D	−0.38*	**0.76** *	0.11	**0.73** *

**M3**					**M3**				

AIS A	0.63				AIS A	0.27*			
AIS B	0.58	**0.99** *			AIS B	0.43*	**0.97** *		
AIS C	0.58	**0.99** *	**1.00** *		AIS C	0.11	**0.94** *	**0.92** *	
AIS D	0.51	**0.88** *	**0.92** *	**0.92** *	AIS D	0.24*	**0.87** *	**0.80** *	**0.78** *

Bold text indicates a strong correlation (*r* ≥ 0.7). Asterisk indicates statistical significance (*p* < 0.05).

**FIGURE 5:**
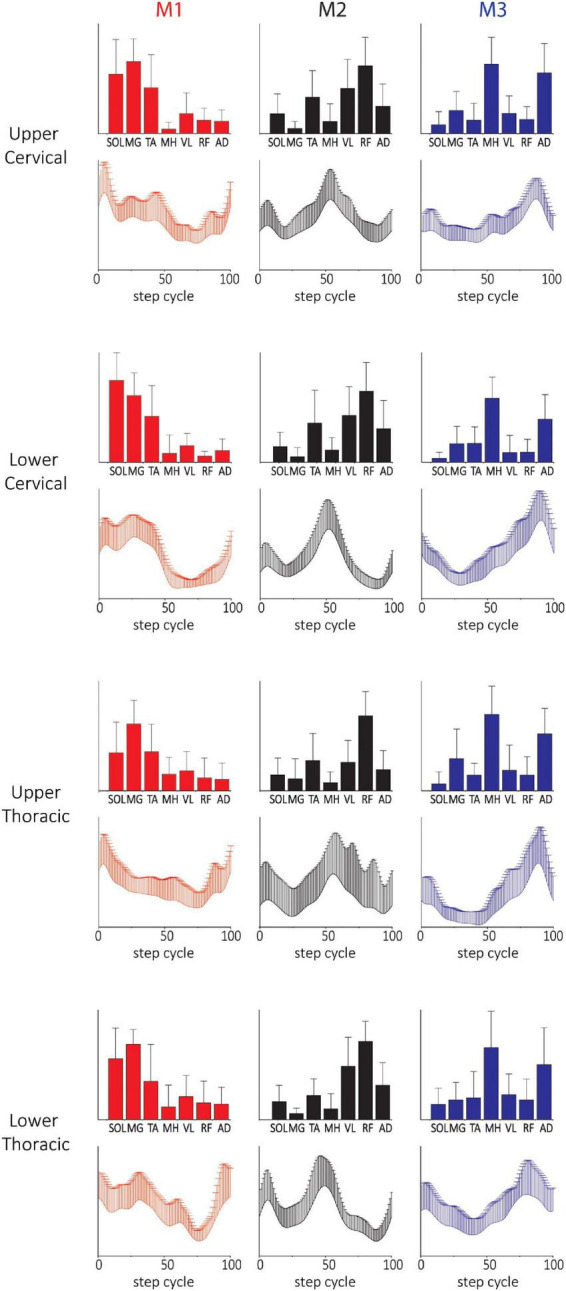
Three-(fixed) modules extracted from SF-NMF sorted by NLI. Each panel shows spatially-fixed synergy patterns grouped by NLI (group mean + SD). The patterns were generally well correlated across NLI categories. See Supplementary Table 3 for details on correlations.

The spatial and temporal patterns from SF-NMF and TF-NMF were also mostly similar across SCIs with different NLI (Figure 5 and Supplementary Figure 7) and varying chronicity of SCI (Supplementary Figure 8). The majority of comparisons between modules across neurological levels of injury (Supplementary Table 3) and chronicity (Supplementary Table 4) indicated strong correlations in spatial and temporal patterns among the subgroups of SCIs, but weak correlations to those of NDs. These findings indicate that the plasticity occurring in the spinal cord circuitry after SCI and the NLI do not strongly affect the modular structure expressed in pattern generation during stepping in this SCI population.

### Characteristics of motor modules using 6 muscles in NDs and SCIs

**FIGURE 6:**
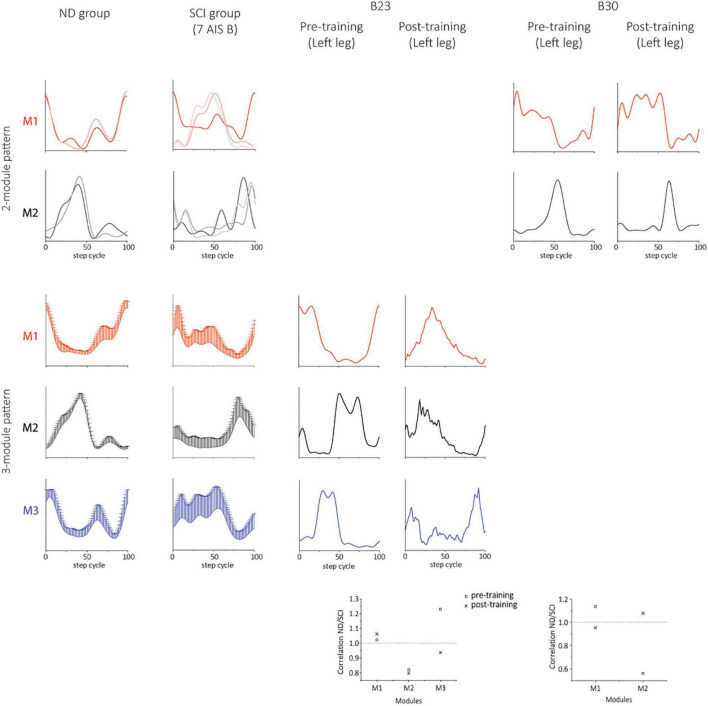
Temporal synergy patterns from SF-NMF using 6 muscles in NDs, SCIs, and two successfully rehabilitated individuals (B23 and B30). Each panel shows temporal patterns from SF-NMF grouped by the number of modules that explain ≥90% variance in the EMG from 6 muscles of each leg. In the 2-module patterns of ND and SCI groups, each line represents a temporal synergy pattern from a leg. The 3-module patterns of ND and SCI groups represent group mean + SD. The scatterplots on the bottom represent the ratio of the maximum absolute correlation of each module. The ratio of greater than 1 indicates that the module exhibits greater similarity to the NDs than to the SCIs. See section “Materials and methods” for further details.

**FIGURE 7:**
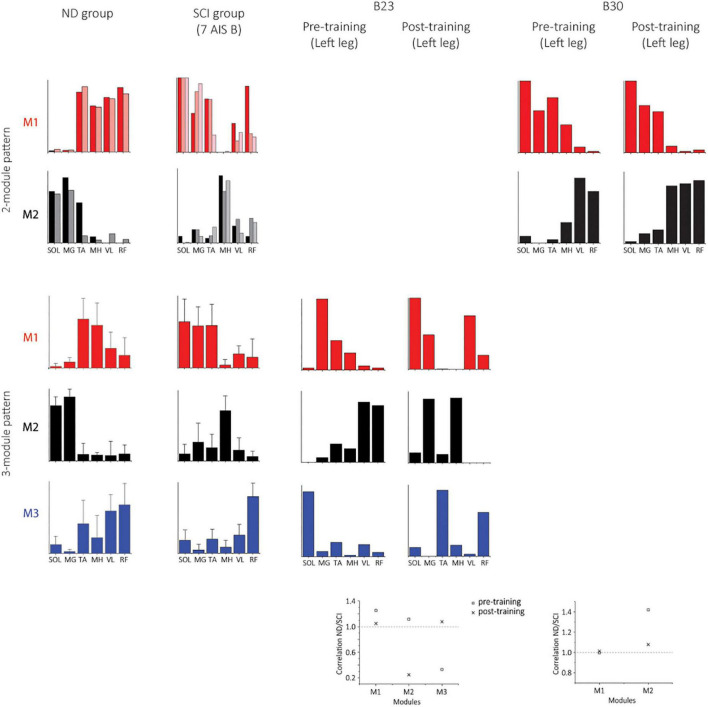
Spatial synergy patterns from SF-NMF using 6 muscles in NDs, SCIs, and two successfully rehabilitated individuals (B23 and B30). Each panel shows spatial patterns from SF-NMF grouped by the number of modules that explain ≥90% variance. In the 2-module patterns of the ND and SCI groups, each bar represents a spatial synergy pattern from a leg. The 3-module patterns of ND and SCI groups represent group mean + SD. The scatterplots on the bottom represent the ratio of the maximum absolute correlation of each module between the ND and SCI group mean synergies (2- or 3-modules) in B23 (3 modules) or B30 (2 modules). The ratio of greater than 1 indicates that the module exhibits greater similarity to the NDs than to the SCIs.

Two subjects (B23 and B30) achieved independent walking in the presence of scES following locomotor training with task-specific stimulation. In order to examine synergy patterns in those two subjects with 6 EMGs (RF, VL, MH, TA, MG, and SOL) collected post-training, we performed the NMF analysis with 6 muscles in the non-ambulatory AIS B SCI subjects (*n* = 7, randomly selected) and NDs (*n* = 7). The number of motor modules extracted from 6 muscles varied from 2 to 4 in NDs and SCIs. In NDs, SF-NMF resulted in 2 modules (2 legs in 2 individuals) and 3 modules (12 legs in 7 individuals, Figures 6, 7 ND group), whereas TF-NMF resulted in 3 modules to explain ≥90% VAF in all individuals (14 legs). The spatial and temporal patterns of three-module synergy were consistent with those extracted from 7 muscles. In SCIs (7 AIS B subjects), SF-NMF yielded 3 modules in 6 individuals (11 legs’ data) and 2 modules in 2 individuals (3 legs’ data; Figures 6, 7, SCI group), and TF-NMF yielded 3 modules in all individuals (12 legs’ data), 2 modules in 2 individuals (2 legs’ data, 1 in each individual).

### Characteristics of motor modules post-training in two successfully rehabilitated AIS B individuals

Post-training, the subject B23 walked on the treadmill at 0.13 m/s and 70% BWL, and B30 walked at 0.72 m/s and 65% BWL, respectively. Twelve concatenated steps from B23 and 36 steps from B30 were analyzed. The number of motor modules was mostly similar pre-training and post-training in both subjects. For B23, SF-NMF using 6 muscles led to 3 modules to explain ≥ 90% VAF pre- and post-training, and TF-NMF led to 4 modules pre- and post-training both legs. For B30, SF-NMF yielded 2 modules to explain ≥ 90% VAF pre- and post-training, and TF-NMF produced 3 modules pre-training and 2 modules post-training for both legs.

Training changed both temporal and spatial synergy patterns. In B23, despite the consistency in the number of modules to explain ≥ 90% VAF pre- and post-training, temporal and spatial patterns of muscle synergies changed (Figures 6, 7). Further, bilateral symmetry increased both for the spatial and temporal patterns. For example, pre-training, Pearson r for the left-right spatial pattern correlation of the three-module patterns ranged 0.15-0.86, and temporal correlation ranged 0.32-0.81 from SF-NMF. Post-training, Pearson r for left-right spatial pattern correlation ranged 0.88-0.92, and temporal pattern correlation ranged 0.75-0.95. In B30, temporal and spatial patterns obtained from SF-NMF were similar pre- and post-training. However, the patterns from TF-NMF had a reduction in motor modules. Bilateral symmetry remained consistently high in B30 pre- and post-training. Pre-training, Pearson r for the left-right spatial pattern correlation of the two-module pattern was 0.99 and 1, and temporal correlation was 0.93 and 0.96 from SF-NMF. Post-training, Pearson r for left-right spatial pattern correlation was 0.97 and 1, and temporal pattern correlation was 0.93 and 0.94. While some of the motor modules changed, the synergy patterns did not exhibit greater similarity to the ND patterns (Figures 6, 7, scatterplots on the bottom), indicating that the recovery of walking function is not dependent on normalized synergy patterns.

## Discussion

In this study, we have characterized the modular structure of the locomotor network that the spinal cord is capable of producing. We identified modularity in response to the step-related sensory feedback received during assisted stepping in subjects with severe SCI, and able-bodied locomotion (NDs). We also compared clinical characteristics, such as AIS, NLI, and YSI to modular control of locomotion and constituent muscle synergies. In addition, we analyzed the changes in synergy patterns in two individuals who achieved independent walking overground with scES, following step training with epidural stimulation (Angeli et al., 2018). Synergy patterns identified by ICA in SCIs mostly presented as single muscle-level synergies, and the NMF analyses were better suited to identify consistent synergy structure for the surface EMGs collected here. Our results indicated that the numbers of synergy patterns identified in SCIs were more variable than those in NDs. Further, neither the severity of the injury, the expected strength of supraspinal inputs, nor the post-injury time available for neuroplastic changes of the spinal circuitry was related to differences in the synergy structures in the SCIs. The synergy modules extracted by SF- and TF-NMF were largely similar within each group (NDs, SCIs). We demonstrated a highly consistent 3-module synergy pattern capturing ≥90% VAF in the ND group in NMF analyses within which M1 and M2 were highly similar but M3 could show more variation with analysis method (SF vs. TF). The SCI synergy characteristics are discussed in detail below. Lastly, our results in two successfully rehabilitated individuals indicated no discernable patterns that were consistent between them, indicating that the recovery of walking function with epidural stimulation is not dependent on the normalization of the spinal generated synergy patterns. This may be due to the fact that independent walking is only achieved during the combination of epidural stimulation and the subject’s intention to walk, and thus may depend highly on neuromodulation and supraspinal controls. Nonetheless, small changes observed in the synergy patterns without stimulation indicate that the interneuronal network that generates synergy patterns in the lumbosacral spinal cord undergoes some changes during the locomotor training and scES, and these changes may also contribute to the recovery.

In the SCI group, SF- and TF-NMF identified 2-5 modules required for ≥90% VAF capture in stepping EMG. Subjects with 2-module synergy patterns, showed a peak of each during the stance or swing phase, similar to the patterns identified during stepping in neonates (Dominici et al., 2011). This resemblance suggests that the expression of the 2-module pattern may be related a collapse of synergies to a de-differentiated pattern caused by insufficient supraspinal control of the locomotor circuitry and compromised propriospinal and proprioceptive control. The spatial and temporal profiles of 3-, 4- and 5-module patterns showed some correlation between healthy modules and SCI modules, but also the SCI coactivation of antagonistic muscles (e.g., SOL, MG, and TA). When taken together, the data suggest that following SCI, even non-collapsed motor modules are expressed differently in some way from healthy modules. These findings further illustrate that the modular control of the locomotor circuitry either reverts to a more primitive structure as suggested by Yang, Logan (Yang et al., 2019), or else alternatively becomes tuned to or reorganized by the feedback controllers or specific biomechanical task requirements following SCI. For example, while commonly considered maladaptive, the coactivation of ankle muscles shown during stepping may be advantageous for tasks such as transfers or limb stabilization in SCIs (Lee et al., 2020).

Interestingly, we found that the synergy structures remained largely stable across AIS, YSI, and NLI. Ivanenko et al. (2003) reported that the motor modules identified from subjects with AIS C and D exhibited greater similarities to modules of NDs. This is inconsistent with our findings, as in 3-module patterns, spatial and temporal patterns of AIS C and D subjects showed a stronger correlation with those of AIS A and B subjects, rather than with those of NDs. This inconsistency may be due to the fact that the subjects in Ivanenko et al. (2003) underwent daily sessions of body weight-supported treadmill training for 1-3 months prior to the recording of leg muscle activity. In contrast, our subjects were untrained in any specific way prior to testing, except for the data from two participants (B23 and B30) presented after training. The training sessions might have helped guide the Ivanenko’s SCI subjects to achieve synergy patterns closer to the healthy modules in AIS C and D subjects (Ivanenko et al., 2003). Further, our results of post-training synergies in two subjects indicate that the synergies sometimes do not strongly correlate with either SCIs or NDs (B23) or else change little and still correlate more to SCI (B30). These results suggest that the propriospinal contribution to the activation of synergies in these subjects post-training with scES is not adequate to normalize the synergy patterns even though the circuitry is capable of generating independent walking when additional excitation is provided to the spinal cord below the lesion. Alternatively, or in addition, it may be that specific supraspinal input is required for the generation of synergies similar to the NDs. Our new data also suggest that the step training combined with neuromodulation in spinal cord injured subjects may re-organize spinal circuitry differently compared to the locomotor training alone.

Our data suggest that AIS is not predictive of the modular control of the locomotor circuitry in response to afferent inputs following SCI. While AIS classification is a widely used diagnostic and classification tool in individuals with SCI, here and elsewhere, it is limited in capturing physiological and functional changes and classifying residual functions and potential for recovery following SCI (Steeves et al., 2007; Krishna et al., 2014; Kirshblum et al., 2021). Further, examining YSI also indicated stronger correlations among groups with different time frames since the injury, suggesting a striking level of stability in motor modules and in the core locomotor circuitry in SCIs over time, despite the reorganization of the spinal cord circuitry clearly occurring after injury (de Leon et al., 1998, 1999; Rossignol et al., 1999, 2008; Hiersemenzel et al., 2000; Pearson, 2000; Schindler-Ivens and Shields, 2000; Rossignol, 2006; Cote et al., 2012; Beauparlant et al., 2013; Houle and Côté, 2013; Tseng and Shields, 2013; Takeoka et al., 2014; Asboth et al., 2018) and regardless of rehabilitation status. This is consistent with findings in the rodent SCI model (Yang et al., 2019), but it contrasts with the previous findings in people post-stroke during upper extremity tasks (Cheung et al., 2012). There, synergy patterns of upper extremity muscles were directly related to the chronicity of stroke (Cheung et al., 2012). Further, a strong correlation among patterns of NLI categories also indicated that the modular control of leg muscles is not strongly influenced by the level of disruption of intersegmental coupling between arms and legs (Bergmans et al., 1973; Miller et al., 1975; English and Lennard, 1982; Muzii et al., 1984; Wannier et al., 2001; Donker et al., 2002). This result also indicates that the autonomic dysfunction present in subjects with T6 injury or above does not influence the modular control nor the plasticity of the locomotor circuitry modularity expression following injury.

Populations with different neuropathologies possess different impairments of modular control of locomotor circuitry. Types of descending inputs lost or disrupted may affect the manifestation of motor modules differently. Individuals post-stroke showed a reduced number and complexity of modules (Clark et al., 2010; Kautz et al., 2011; Routson et al., 2014) due to merging of motor modules (Clark et al., 2010), suggesting that cortical lesion leads to the inability to independently activate each module (Clark et al., 2010; Safavynia et al., 2011). Similarly, in people with Parkinson’s disease, fewer motor modules were identified compared to healthy subjects, and temporal patterns were altered while some spatial synergy patterns were unaffected (Rodriguez et al., 2013). Individuals with cerebellar lesions showed similar numbers of modules to healthy subjects with an altered duration of muscle activation in the temporal pattern (Martino et al., 2015). While some of our results showed similarities to the previous findings regarding fewer motor modules compared to healthy subjects (Fox et al., 2013; Hayes et al., 2014; Danner et al., 2015; Perez-Nombela et al., 2017), we also identified similar and greater complexities in motor modules in some SCI subjects. These differences may be attributable to the ambulatory capabilities of individuals with SCI tested and the conditions in which the locomotor patterns were induced. The supraspinal control of the locomotor circuitry and the use of assistive devices may together contribute to a constrained and simplified modular expression (Fox et al., 2013; Hayes et al., 2014; Perez-Nombela et al., 2017). The varying number of motor modules, as well as altered temporal and spatial patterns in the SCI subjects in our study, suggests that the loss of supraspinal inputs in the SCI subjects has extensive effects on spinal rhythm and pattern generation circuitry, and that these deficits of the modular control are not easily characterized by the commonly used clinical characteristics such as YSI, AIS, and NLI.

Our results on laterality illustrated that there was asymmetry in the number of modules, but, unsurprisingly, no inherent sidedness. While exploring bilateral symmetry of synergy patterns was not within the scope of this study, the rhythm and pattern generation between the two sides are clearly not independent of each other based on simulation and experimental investigations of the locomotor central pattern generator (Lanuza et al., 2004; Rybak et al., 2006, 2015; Crone et al., 2008; McCrea and Rybak, 2008; Dougherty et al., 2013; Hagglund et al., 2013; MacLellan et al., 2014; Ha and Dougherty, 2018; Dougherty and Ha, 2019). Further, greater bilateral symmetry in post-training synergies in one of the subjects (B30) may indicate that the recovery of bilateral symmetry is associated with the recovery of walking with neuromodulation. Future work may explore whether and how the synergy characteristics expressed on one side influence the synergy characteristics on the other side in the SCI stepping systems.

In SCI subjects, despite findings from Ivanenko et al. (2003), it is still unclear whether rehabilitation strategies can improve the modular control of locomotor circuitry across the range of SCI population and/or lead to modular characteristics more similar to NDs. Our data here, when combined with the earlier Ivanenko study, suggest that treatment modalities and rehabilitation combinations may have major impacts on this. Post-stroke, both merging and fractionation of muscle synergies during walking were observed over the course of therapy (Hashiguchi et al., 2016), and both spatial and temporal patterns of modularity can become similar to the healthy pattern after gait training (Routson et al., 2013). In SCI rats, electrical stimulation of motoneuron pools that mimics the spatiotemporal activation of muscle synergies seen during locomotion in healthy rats could improve weight-bearing ability and quality of locomotion (Wenger et al., 2016). As innovative techniques such as epidural and transcutaneous electrical stimulation combined with gait training and pharmacotherapy develop, it will be crucial to further investigate how these neuromodulatory techniques impact modular control of locomotor circuitry and generation of synergy patterns. It is of particular interest whether features of spinal-generated modular patterns, e.g., the number of modules or specific synergy patterns, serve as constraints or predictors for functional improvement.

There are two competing ways to think of the origins of synergies in locomotion: In the first locomotion synergies are relatively fluid and may be emergent from the combined influences of load and kinematic feedback systems of spinal cord operating as an ensemble under, or separated from descending control; in the second locomotion synergies may be evolutionarily pre-determined and have spinal structural bases, but be actively adjusted or augmented through reflex and descending control in voluntary tasks. Given the range of SCI subjects tested pre-training, we believe our data are consistent with a common core infrastructure of synergies, expressed in all SCI subjects despite their wide variations in condition. However, it remains conceivable that if the complex and technically difficult experiment were to be performed in which the initial untrained SCI kinematics and loading in stepping were somehow precisely matched to some variant of identical ND kinematics and loading, the patterns in stepping and the synergy production of each would converge to identical outputs. In the absence of such a technical tour de force experiment, we favor the hypothesis of a common synergy infrastructure in spinal cord across all SCI subjects, modified in intact individuals through voluntary and other descending controls of spinal cord. It is noteworthy that both perspectives on synergy origins expect that some SCI subjects will achieve good stepping and weight support after extensive training, as described in Grasso et al. (2004) where detailed kinematics and EMG analyses were combined with the modular EMG organization presented in Ivanenko et al. (2003). Presumably, this training outcome must build on and further engage the consistent synergy patterns described here in the non-ambulatory initial conditions.

The purpose of manual facilitation during stepping was to provide stepping-related sensory feedback that provides excitation to the spinal circuitry that is otherwise silent, and to optimize body kinematics. Further, in the SCIs, step speed and percent BWL were selected for each subject to produce the optimal stepping pattern and muscle activation with manual facilitation. However, the excitation that the selected step speed and BWL provided to the spinal cord may not be identical to the excitation in NDs, who did not require manual facilitation during stepping.

In conclusion, we demonstrated that SCI synergy patterns expressed were mostly stable across different severities of injury, strength of supraspinal inputs (AIS grade), and length of time, and also largely similar among SCI subjects, though differing from NDs. Further, the recovery of walking function following spinal cord epidural stimulation and locomotor training does not lead to the normalization of synergy patterns during stepping without the scES in two AIS B subjects. We believe that our findings provide a basis for future investigations of neural and biomechanical mechanisms underlying the modular organization of locomotor circuitry and constraints on plastic changes, and gait symmetry during training in people with SCI. In addition, the consistent synergy patterns in the SCI subjects and their variations from healthy patterns must impact the design and assessment of future rehabilitation strategies.

## Data availability statement

The raw data supporting the conclusions of this article will be made available by the authors, without undue reservation.

## Ethics statement

The studies involving human participants were reviewed and approved by the Institutional Review Board at the University of Louisville. The patients/participants provided their written informed consent to participate in this study.

## Author contributions

SYS and CAA designed research. SJH and CAA collected the data. SYS, SFG, and CAA analyzed the data. SFG helped design and data analysis. SYS, SFG, and CAA wrote the manuscript. All authors contributed to the article and approved the submitted version.
