# Supplementary material for: Modular organization of locomotor networks in people with severe spinal cord injury
Source: Front Neurosci. 2022 Dec 7;16:1041015. doi: 10.3389/fnins.2022.1041015 (PMC9768556; doi:10.3389/fnins.2022.1041015)
Supplement: Supplementary file 1 [file Data_Sheet_1.docx]

**Supporting Information (SI)**

The Supporting Information includes:

Supplementary text (Results)

Figures S1 to S8

Tables S1 to S4

**SI Results**

*Breaking points or “elbow” in percentage VAF in ICA*

To examine if the %VAF breaking points coincided with the number of motor modules extracted from the ICA, piecewise linear regression was used. Results indicated that breaking points identified by piecewise regression ranged 2-4 modules in NDs and 2-6 modules in SCIs, and the piecewise regression identified fewer modules than the VAF criterion both in NDs (*df = 13, t = -8.92, p < 0.001;* Fig. S3A) and SCIs (*df = 145, t = -23.79, p < 0.001;* Fig. S3B). Further, in NDs, a repeated-measures ANOVA indicated that the number of modules from the SF-NMF, TF-NMF, and piecewise regression of ICA VAF were similar, *F(2, 26) = 0.38, p > 0.6.* In contrast, in SCIs, the number of modules from the 3 analyses was significantly different, *F(2, 286) = 25.27, p < 0.001*, and post hoc comparisons indicated that the number of modules was all significantly different from each other (*Bonferroni* *p<0.02*).

*Synergy Patterns with Correlation of < 0.7*

In TF NMF, 4 out of 38 patterns (10.53%) were excluded in 2-module patterns, 26 of 255 patterns (10.20%) were excluded in 3-module patterns, and 12 out of 140 patterns (8.57%) were excluded in 4-module patterns because correlations were < 0.7. In 5-module TF NMF patterns, 4 out of 35 patterns (11.43%) were excluded. The correlation of the temporal patterns between the excluded dataset and presented dataset ranged from -0.46 to 0.26 (-0.05±0.29) in the 2-module pattern, -0.72 to 0.55 (-0.03±0.55) in the 3-module pattern, -0.61 to 0.60 (-0.02±0.34) in the 4-module pattern, and -0.42 to 0.43 (-0.12±0.25) in the 5-module pattern.

In SF NMF, 5 out of 64 patterns (7.81%) were excluded in 2-module patterns, 28 of 291 patterns (9.62%) were excluded in 3-module patterns, and 8 out of 68 patterns (11.76%) were excluded in 4-module patterns. The correlation of spatial patterns between the excluded dataset and presented dataset ranged from -0.36 to 0.53 (0.02±0.37) in the 2-module pattern, -0.76 to 0.62 (-0.01±0.30) in the 3-module pattern, and -0.49 to 0.60 (-0.01±0.30) in the 4-module pattern.

The majority of the patterns that showed a lower correlation coefficient was due to the muscles that were tonically active or inactive; therefore, we determined that they were not meaningful in interpreting synergies. Other patterns among the < 0.7 correlations were highly inconsistent and did not show any discernable pattern upon visual examination, so we did not present them in the manuscript.

*Characteristics of motor modules identified by ICA in NDs and SCIs and correspondences to NMF modules*

ICA required a greater number of modules to explain ≥ 90% VAF in the EMG during stepping. We further examined the modular structures extracted from the ICA by investigating the identified muscle weightings. In NDs, the M1 and M2 showed muscle weightings that were similar to the spatial patterns in M1 and M2 extracted from SF- and TF-NMF. For example, Fig. S5A demonstrates that while 5 modules were required to explain ≥ 90% VAF, M1 consisted of TA, MH, VL, RF, and AD activity, and M2 consisted of SOL and MG activity. Comparable muscle weightings were observed in all ND subjects (e.g., Fig. S5B). When correspondences to the spatial patterns from SF- and TF-NMF were examined using correlation, results indicated that M1 from the ICA, when averaged across NDs, was highly correlated to the M1 from SF-NMF and TF-NMF (Pearson r = 0.99 for both), and M2 from the ICA was highly correlated with M2 and M3 in SF-NMF (r = 0.71 and 0.76, respectively) and M2 in TF-NMF (r = 0.98), but was only moderately correlated with the TF-NMF M3 (r=0.55).

In contrast, in SCIs, ICA often resulted in modules that were primarily based on a single muscle. As such, it is understandable that those components accounted for small percentage of VAF and did not present a clear breaking point in visualization of the cumulative VAF. For example, the muscle weighting in a subject with T4 ASI A SCI (Fig. S5C) showed that while M1 identified a synergistic activity of SOL and VL, other modules were based on one muscle (M2: MG, M3: AD, M4: MH, M5: TA). Muscle weighting identified in a subject with C7 ASI D SCI showed a similar pattern (Fig. S5D). These results indicated no clear synergistic pattern extracted by the ICA regardless of NLI and severity of the injury, suggesting that the NMF analysis is more suitable in identifying the motor modules in people with SCI given the number of muscles and EMG signal characteristics.

**
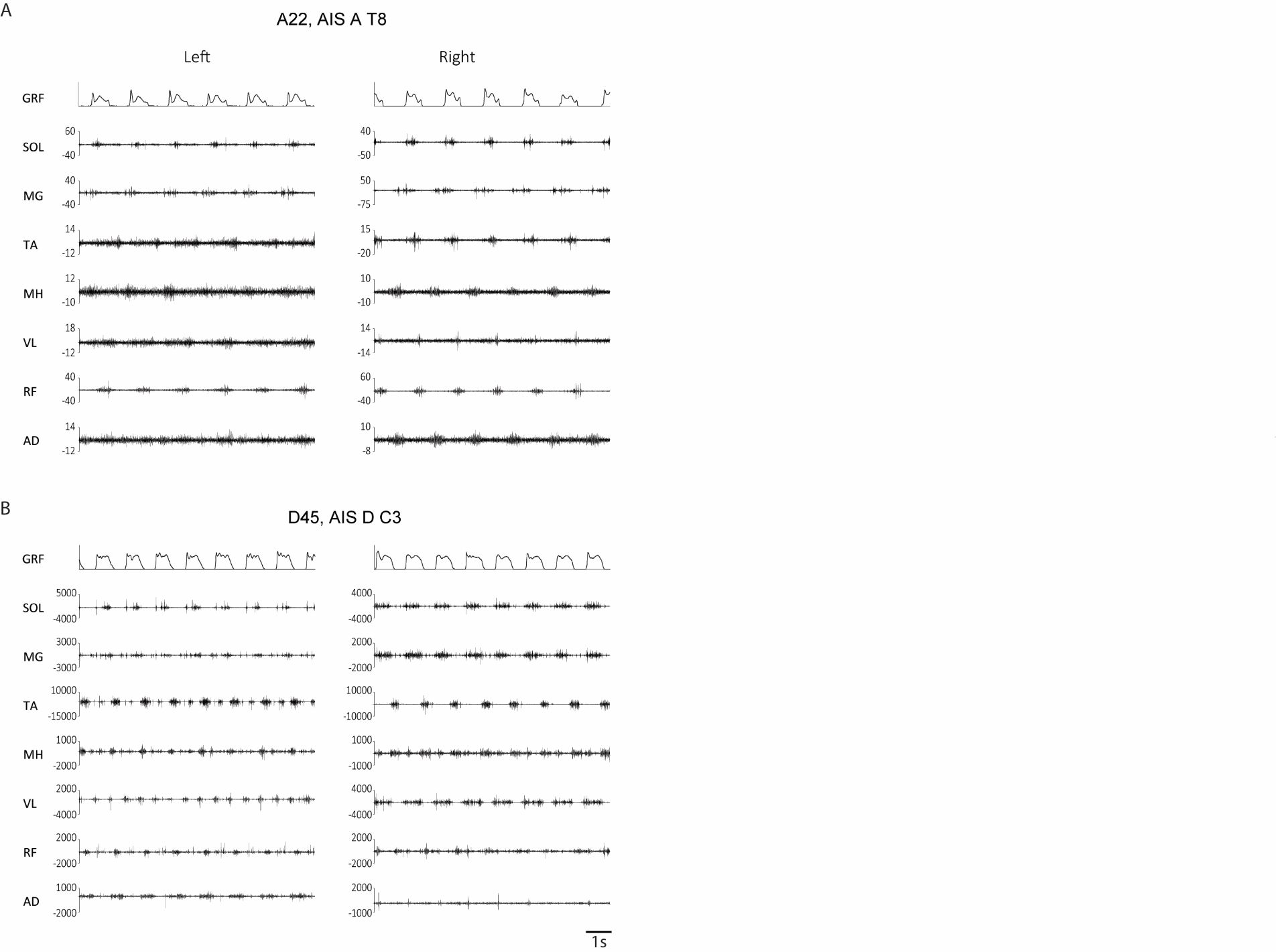
**

**Supplementary Figure 1.** **Patterns of EMG during stepping.**

EMG traces are shown for 14 muscles bilaterally; left and right soleus (SOL), medial gastrocnemius (MG), tibialis anterior (TA), medial hamstring (MH), vastus lateralis (VL), rectus femoris (RF), and adductor (AD). Ground reaction force traces (GRF) are shown on top. **A**. EMG traces from subject A22, who had T8 AIS A injury (YSI = 2.1 yrs). The subject was stepped at 46% BWL and 0.98 m/s. All of the muscles show rhythmic activity, even though the amplitude of the muscle activity is smaller, as indicated by y-axis of each trace (in V). **B**. EMG traces from subject D45, who had C3 AIS D injury (YSI = 0.8 yrs). The subject was stepped at 75% BWL and 1.07 m/s. The amplitude of all muscle activities is greater than the subject in **A**. Horizontal scale equals 1 second.

**
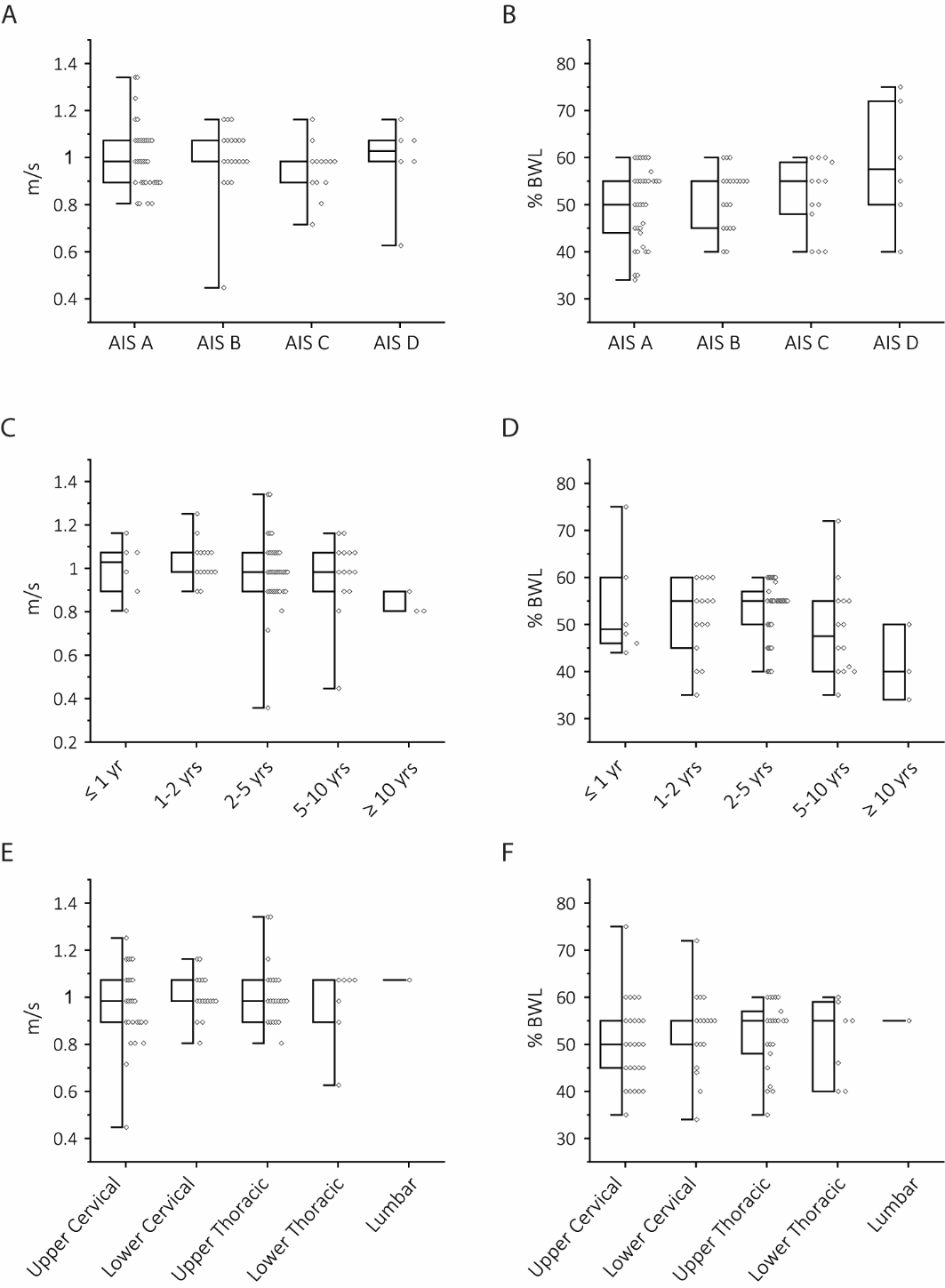
**

**Supplementary Figure 2.** **Step speed and % body weight load (%BWL) during SCI stepping.**

The box and scatter plots indicate step speed and % body weight load (BWL) to produce optimal kinematic and EMG patterns during stepping with manual facilitation on the treadmill. Step speed and % body weight load (BWL) that produced optimal stepping patterns varied across subjects. The mean (±SD) step speed was 0.99 ± 0.14 m/s (range: 0.45-1.34 m/s), and the mean (±SD) BWL was 51.66 ± 8.88% (range: 34-80%). Each box indicates 25 percentile and 75 percentile, and the horizontal line in the box indicates a median value. Whiskers indicate the minimum and maximum values. Each dot indicates an individual data point. **A-B.** Step speed (**A**) and %BWL (**B**) categorized by AIS (34 AIS A subjects, 20 AIS B, 13 AIS C, 6 AIS D) **C-D.** Step speed (**C**) and %BWL (**D**) categorized by YSI (≤ 1 yr since the injury n = 6 subjects, 1-2 yrs since the injury n = 15, 2-5 yrs since the injury n = 35, 5-10 yrs since the injury n = 14, > 10 yrs since the injury n = 3). **E-F.** Step speed (**E**) and %BWL (**F**) categorized by NLI (26 subjects with upper cervical injury, 17 lower cervical injury, 22 upper thoracic injury, 7 lower thoracic injury, and 1 lumbar injury). Statistical analysis indicated that these stepping parameters were similar across clinical characteristics.

**
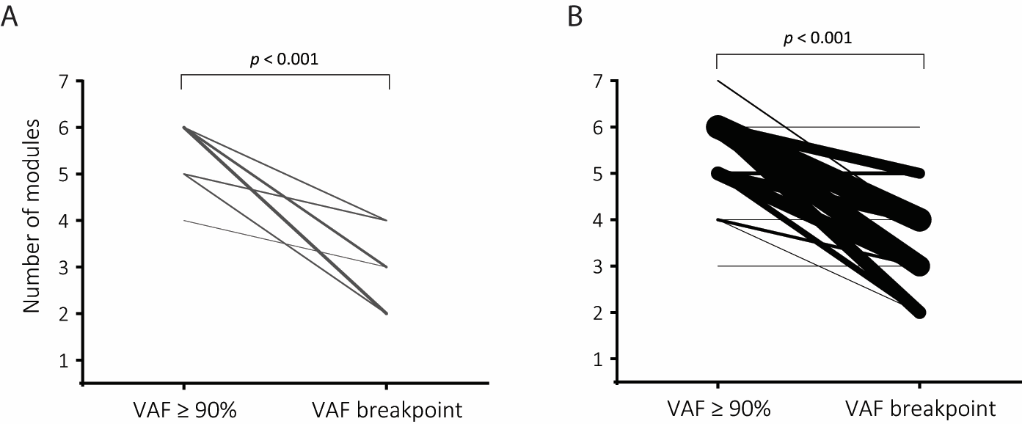
**

**Supplementary Figure 3.** **Comparison of the number of modules identified by ≥ 90% VAF criterion and a piecewise linear regression identified a breakpoint in VAF.**

Each line represents a comparison of the number of modules in each leg. Piecewise linear regression on the VAF resulted in significantly fewer modules than the ≥ 90% VAF criterion both in NDs (**A**) and SCIs (**B**). The thicker the lines, the greater prevalence of the pair.

**
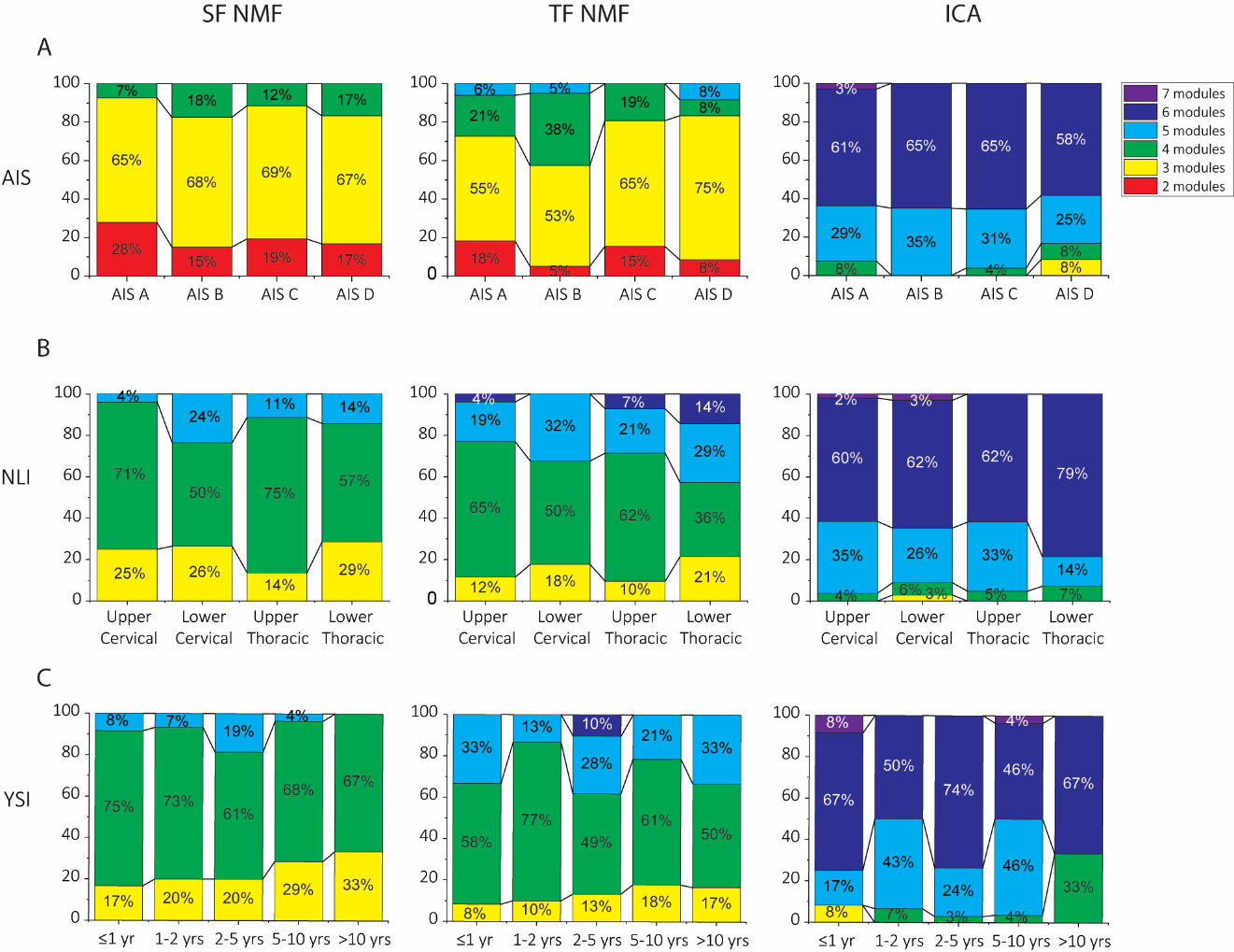
**

**Supplementary Figure 4.** **Distribution of the number of modules across clinical characteristics.**

Percent distributions of the number of modules extracted by SF-NMF, TF-NMF, and ICA sorted by AIS (**A**), NLI (**B**), and YSI (**C**). The distribution of the number of modules was similar across clinical characteristics.


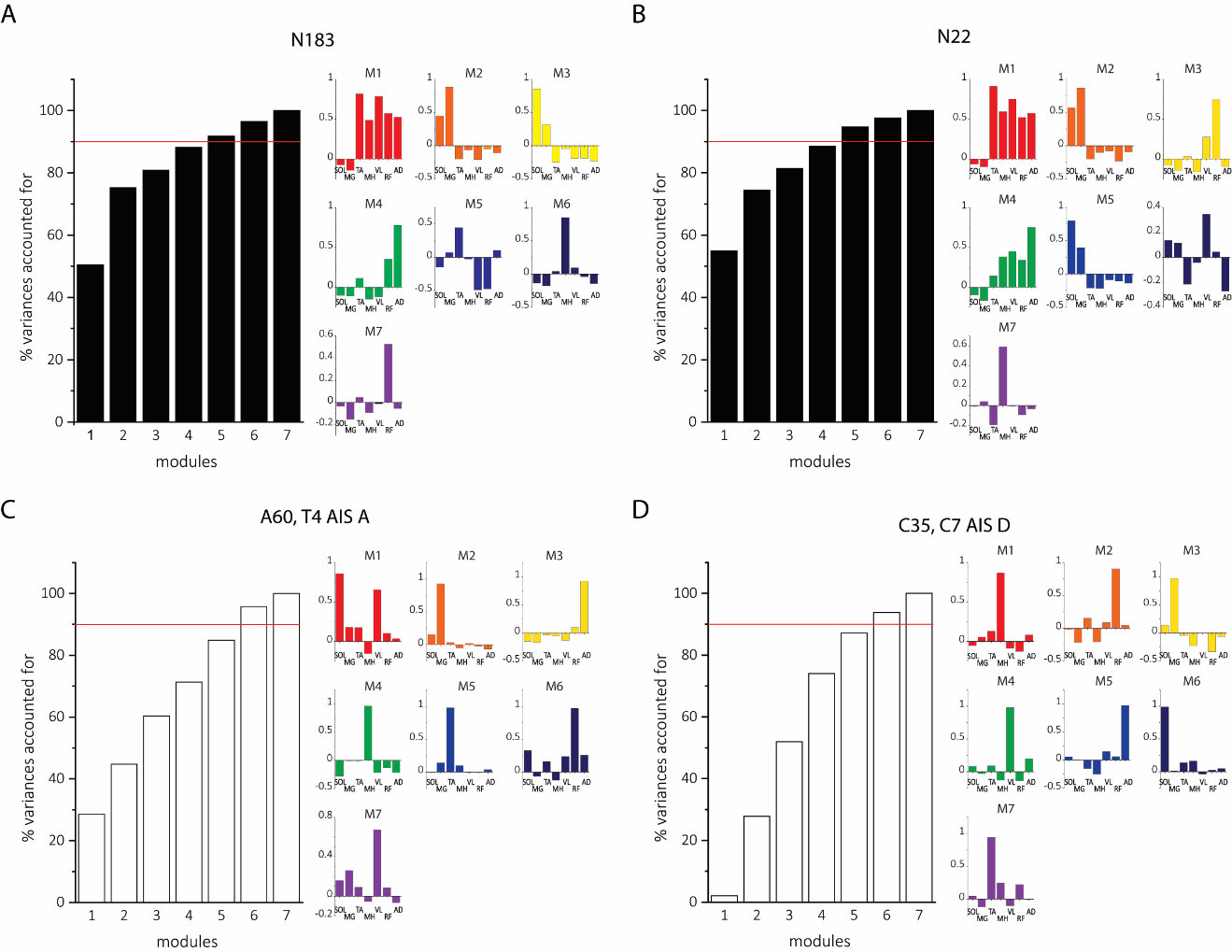


**Supplementary Figure 5.** **%VAF and muscle weighting (spatial) pattern identified by ICA in NDs and SCIs.**

Exemplary ICA results in the NDs (**A** and **B**) and SCIs (**C** and **D**). Within each panel, the bar graph on the left indicates %VAF, and the smaller bar graphs on the right represent the muscle weighting of each component. **A.** Motor modules of the left leg of an ND subject. While 5 modules were required to explain 90% VAF, M1 shows synergistic activity between TA, MH, VL, RH, and AD, and M2 and M3 show synergistic activity of SOL and MG. **B.** Motor modules of the left leg of another ND subject. M1 and M2 showed similar muscle synergy patterns as M1 and M2 in **A**. **C.** Motor modules of the right leg of an SCI subject (A60), who sustained a T4 ASI A injury. **D.** Motor modules of the left leg of another SCI subject (C35) with C7 AIS D injury. In **C** and **D**, 6 modules were required to explain ≥ 90% VAF, and each component indicated no clear muscle synergistic patterns. The horizontal red line indicates 90% VAF.

**
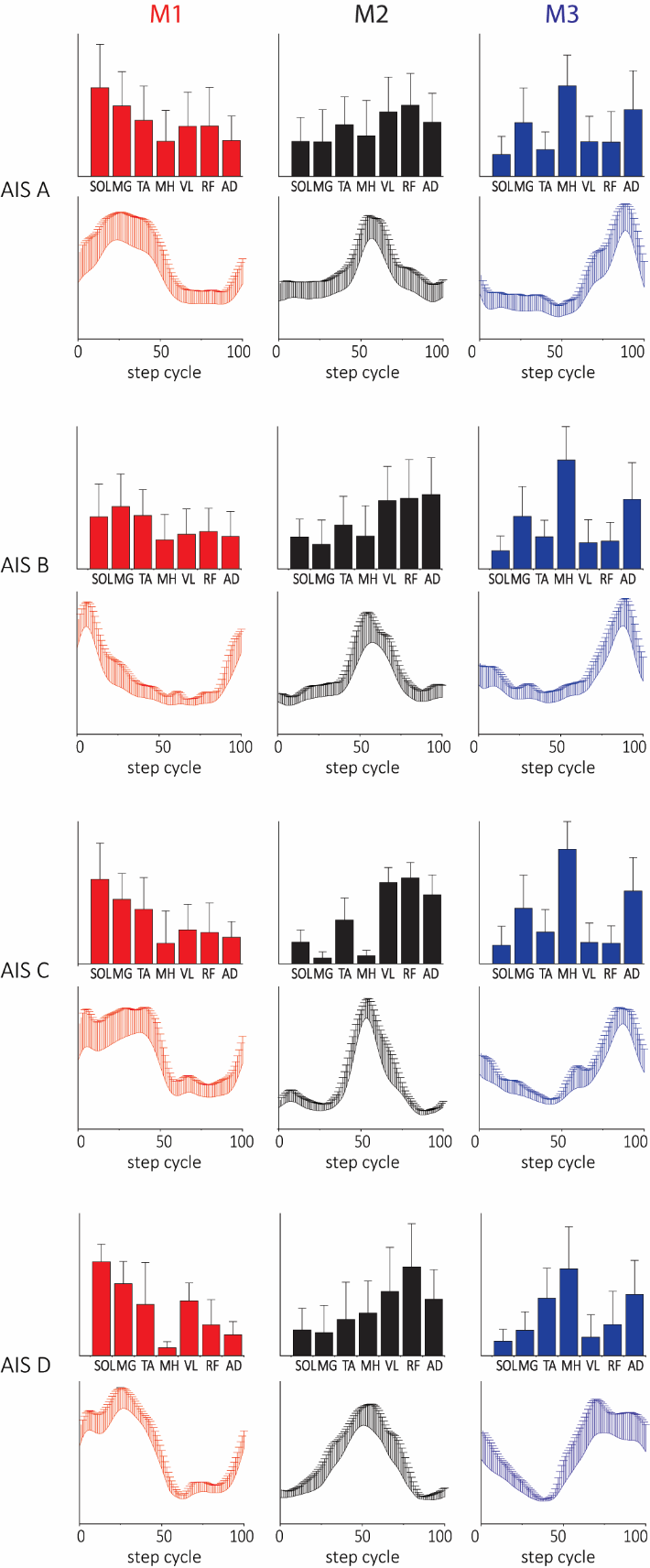
**

**Supplementary Figure 6. Three-(fixed) modules from TF-NMF sorted by AIS.**

Each panel shows temporally-fixed synergy patterns, categorized by AIS (group mean + SD). The patterns were generally well correlated across AIS categories. See Table S2 for details on correlations.


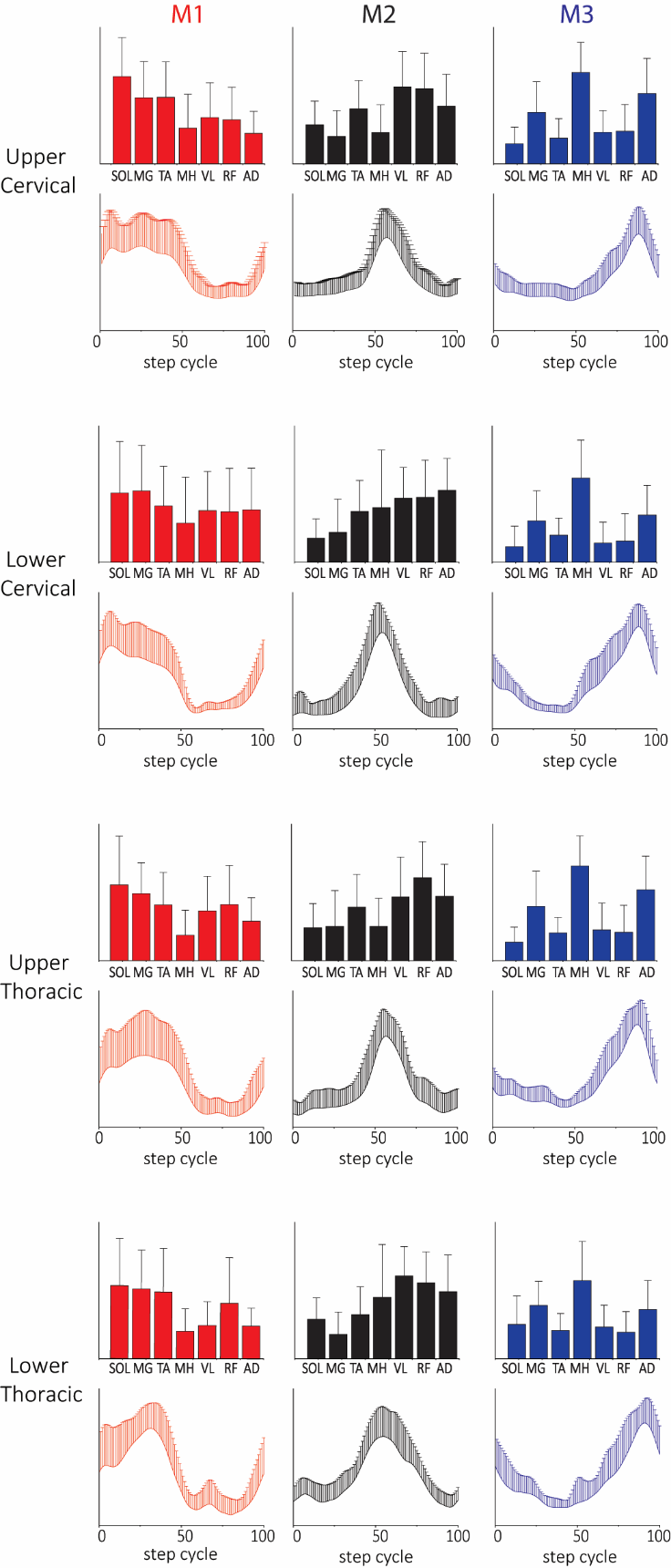


**Supplementary Figure 7. Three-(fixed) modules from TF-NMF sorted by NLI.**

Each panel shows temporally-fixed synergy patterns grouped by NLI (group mean + SD). The patterns were generally well correlated across NLI categories. See Table S3 for details on correlations.

**
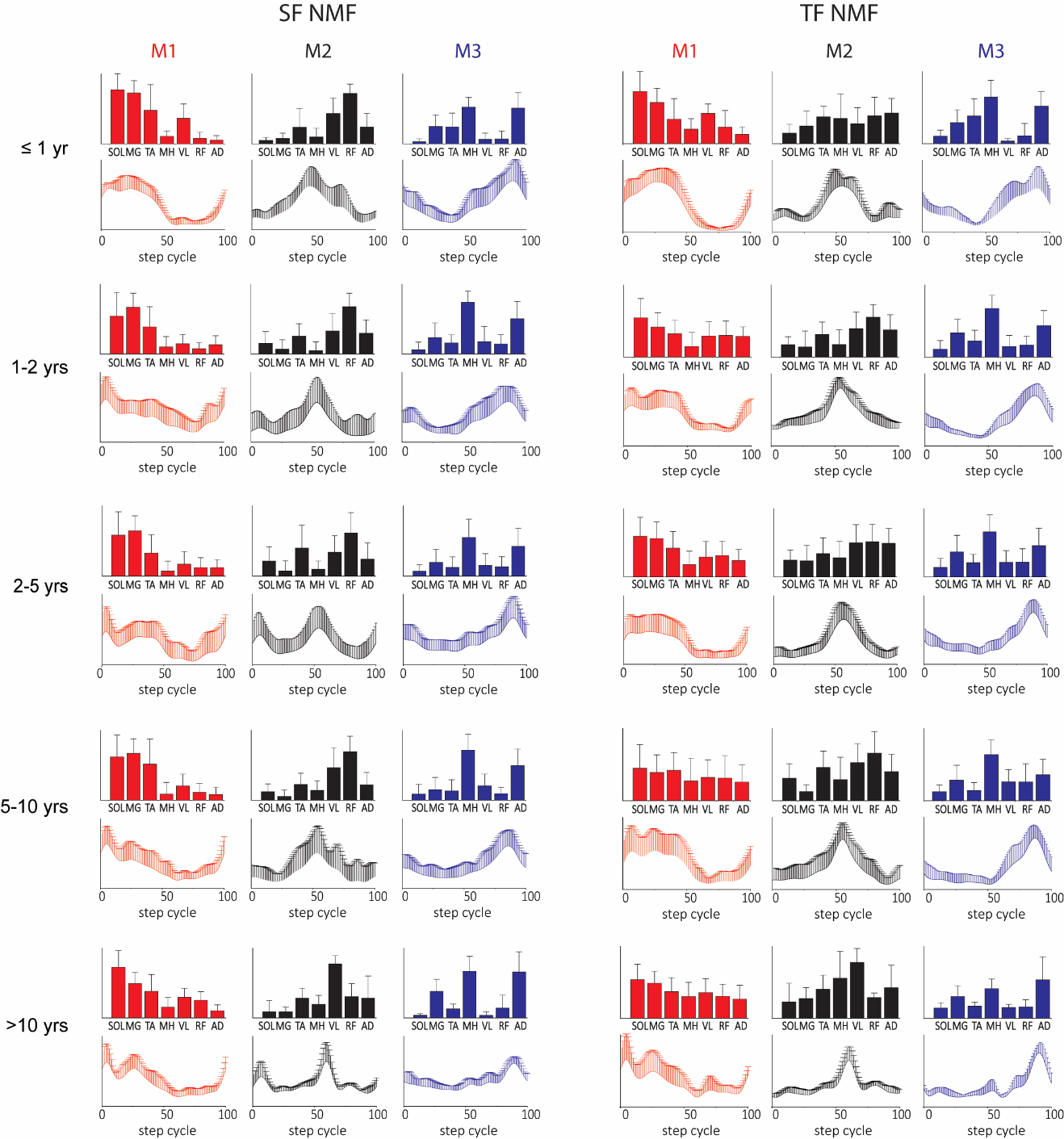
**

**Supplementary Figure 8. Three modules from SF-NMF and TF-NMF, sorted by YSI.**

Figures represent spatially-fixed synergy patterns (left panel) and temporally-fixed patterns (right panel) in SCIs grouped separately by YSI (group mean + SD). The patterns were generally well correlated across YSI categories. See Table S4 for details on correlation.

**Supplementary Table 1. Clinical characteristics of SCI participants**

| AIS A | | | | | AIS B | | | | | AIS C | | | | |
| --- | --- | --- | --- | --- | --- | --- | --- | --- | --- | --- | --- | --- | --- | --- |
| ID | Age | Gender | YSI | NLI | ID | Age | Gender | YSI | NLI | ID | Age | Gender | YSI | NLI |
| A91 | 22 | F | 3.0 | C2 | B26 | 20 | F | 2.6 | C3 | C40 | 35 | M | 9.8 | C2 |
| A37 | 25 | M | 8.4 | C2 | B34 | 26 | M | 2.0 | C4 | A86 | 29 | M | 7.6 | C3 |
| A33 | 51 | M | 1.7 | C3 | B22 | 51 | M | 2.6 | C4 | B06 | 37 | F | 1.1 | C4 |
| A68 | 34 | M | 2.5 | C3 | B16 | 61 | M | 4.7 | C4 | C31 | 24 | F | 1.9 | C4 |
| A41 | 21 | M | 4.3 | C3 | A21 | 30 | M | 4.9 | C4 | C50 | 19 | F | 2.8 | C4 |
| A95 | 30 | F | 6.5 | C3 | B21 | 30 | M | 5.9 | C4 | C20 | 56 | M | 1.4 | C5 |
| A89 | 19 | F | 1.0 | C4 | B29 | 40 | F | 9.2 | C4 | B28 | 37 | M | 7.1 | C6 |
| A67 | 30 | F | 3.8 | C4 | B23 | 31 | M | 2.7 | C5 | C09 | 26 | M | 1.7 | C7 |
| A90 | 66 | M | 5.0 | C4 | B32 | 57 | M | 3.5 | C5 | C49 | 35 | M | 2.8 | C7 |
| A80 | 33 | F | 7.6 | C4 | B27 | 22 | M | 3.5 | C6 | C37 | 20 | F | 6.8 | T2 |
| A34 | 41 | F | 8.8 | C4 | C48 | 31 | M | 3.6 | C6 | C04 | 21 | M | 0.5 | T6 |
| A78 | 30 | M | 12.7 | C4 | B24 | 21 | M | 1.9 | C7 | C47 | 64 | M | 7.2 | T6 |
| A64 | 49 | M | 31.6 | C4 | B13 | 32 | M | 3.0 | C7 | C01 | 45 | M | 3.0 | T8 |
| A20 | 25 | M | 0.7 | C5 | B35 | 30 | M | 2.3 | C8 | **AIS D** | | | | |
| A19 | 65 | M | 14.7 | C7 | B33 | 22 | F | 2.1 | T1 |  |  |  |  |  |
| A69 | 28 | M | 3.4 | C8 | B30 | 22 | F | 2.5 | T1 | D45 | 72 | M | 0.8 | C3 |
| A93 | 28 | M | 4.4 | T2 | B07 | 22 | M | 1.2 | T2 | D11 | 38 | M | 0.5 | C5 |
| A57 | 26 | F | 3.3 | T3 | B20 | 27 | M | 4.2 | T2 | D06 | 35 | M | 10.0 | C6 |
| A82 | 33 | M | 3.7 | T3 | C44 | 40 | M | 1.6 | T4 | C35 | 21 | M | 1.3 | C7 |
| A70 | 32 | M | 4.0 | T3 | C11 | 31 | M | 1.2 | L2 | D13 | 59 | F | 2.1 | T10 |
| A88 | 52 | M | 7.6 | T3 |  |  |  |  |  | D43 | 21 | F | 2.6 | T12 |
| A45 | 23 | M | 1.4 | T4 |  |  |  |  |  |  |  |  |  |  |
| A59 | 26 | M | 2.1 | T4 |  |  |  |  |  |  |  |  |  |  |
| A24 | 42 | M | 2.1 | T4 |  |  |  |  |  |  |  |  |  |  |
| A60 | 22 | M | 2.3 | T4 |  |  |  |  |  |  |  |  |  |  |
| A53 | 28 | M | 1.7 | T5 |  |  |  |  |  |  |  |  |  |  |
| A72 | 26 | M | 5.0 | T5 |  |  |  |  |  |  |  |  |  |  |
| A87 | 23 | M | 1.7 | T6 |  |  |  |  |  |  |  |  |  |  |
| A61 | 48 | M | 2.0 | T6 |  |  |  |  |  |  |  |  |  |  |
| A23 | 27 | F | 7.4 | T6 |  |  |  |  |  |  |  |  |  |  |
| A94 | 35 | M | 4.7 | T7 |  |  |  |  |  |  |  |  |  |  |
| A22 | 28 | M | 0.8 | T8 |  |  |  |  |  |  |  |  |  |  |
| A92 | 38 | M | 3.0 | T8 |  |  |  |  |  |  |  |  |  |  |
| A55 | 36 | M | 4.9 | T9 |  |  |  |  |  |  |  |  |  |  |

YSI = years since injury, NLI = neurological level of injury.

**Supplementary Table 2. Correlation of TF-NMF 3-module patterns among ND and AIS groups**

| Correlation of spatial patterns | | | | |  | Correlation of temporal patterns | | | | |
| --- | --- | --- | --- | --- | --- | --- | --- | --- | --- | --- |
| M1 | ND | AIS A | AIS B | AIS C |  | **M1** | ND | AIS A | AIS B | AIS C |
| AIS A | -0.75 |  |  |  |  | AIS A | -0.22* |  |  |  |
| AIS B | -0.67 | **0.80*** |  |  |  | AIS B | **0.85*** | 0.25* |  |  |
| AIS C | -0.75 | **0.97*** | **0.86*** |  |  | AIS C | -0.05 | **0.96*** | 0.40* |  |
| AIS D | -0.67 | **0.96*** | **0.79*** | **0.94*** |  | AIS D | 0.03 | **0.95*** | 0.49* | **0.97*** |
| M2 |  |  |  |  |  | **M2** |  |  |  |  |
| AIS A | -0.72 |  |  |  |  | AIS A | -0.06 |  |  |  |
| AIS B | -0.70 | **0.89*** |  |  |  | AIS B | -0.06 | **0.98*** |  |  |
| AIS C | -0.64 | **0.95*** | **0.96*** |  |  | AIS C | 0.10 | **0.94*** | **0.91*** |  |
| AIS D | -0.68 | **0.94*** | **0.86*** | **0.87*** |  | AIS D | 0.44* | **0.85*** | **0.85*** | **0.89*** |
| M3 |  |  |  |  |  | **M3** |  |  |  |  |
| AIS A | 0.12 |  |  |  |  | AIS A | **0.81*** |  |  |  |
| AIS B | 0.26 | **0.98*** |  |  |  | AIS B | **0.75*** | **0.98*** |  |  |
| AIS C | 0.26 | **0.97*** | **1.00*** |  |  | AIS C | **0.80*** | **0.97*** | **0.99*** |  |
| AIS D | 0.69 | **0.76*** | **0.85*** | **0.84*** |  | AIS D | **0.87*** | **0.80*** | **0.77*** | **0.82*** |

Bold text indicates a strong correlation (r≥0.7). Asterisk indicates statistical significance (*p*<0.05).

**Supplementary Table 3. Correlation of SF- and TF-NMF 3-module patterns among ND and NLI categories**

| A. SF-NMF analysis | | | | | | | | | | |
| --- | --- | --- | --- | --- | --- | --- | --- | --- | --- | --- |
| Correlation of spatial patterns | | | | |  | **Correlation of temporal patterns** | | | | |
| M1 | ND | Upper Cervical | Lower Cervical | Upper Thoracic |  | **M1** | ND | Upper Cervical | Lower Cervical | Upper Thoracic |
| Upper Cervical | **0.87*** |  |  |  |  | Upper Cervical | 0.26* |  |  |  |
| Lower Cervical | **0.87*** | **0.95*** |  |  |  | Lower Cervical | 0.60* | **0.83*** |  |  |
| Upper Thoracic | **0.82*** | **0.94*** | **0.83*** |  |  | Upper Thoracic | -0.10 | **0.91*** | **0.64*** |  |
| Lower Thoracic | **0.94*** | **0.98*** | **0.94*** | **0.95*** |  | Lower Thoracic | 0.35* | **0.77*** | **0.82*** | **0.74*** |
| M2 |  |  |  |  |  | **M2** |  |  |  |  |
| Upper Cervical | **0.89*** |  |  |  |  | Upper Cervical | 0.17 |  |  |  |
| Lower Cervical | **0.93*** | **0.99*** |  |  |  | Lower Cervical | -0.01 | **0.94*** |  |  |
| Upper Thoracic | **0.81*** | **0.93*** | **0.92*** |  |  | Upper Thoracic | 0.26* | **0.95*** | **0.82*** |  |
| Lower Thoracic | **0.82*** | **0.96*** | **0.96*** | **0.91*** |  | Lower Thoracic | 0.12 | **0.80*** | **0.91*** | 0.65* |
| M3 |  |  |  |  |  | **M3** |  |  |  |  |
| Upper Cervical | 0.62 |  |  |  |  | Upper Cervical | 0.20* |  |  |  |
| Lower Cervical | 0.57 | **0.96*** |  |  |  | Lower Cervical | 0.36* | **0.95*** |  |  |
| Upper Thoracic | 0.57 | **0.98*** | **0.97*** |  |  | Upper Thoracic | 0.31* | **0.98*** | **0.98*** |  |
| Lower Thoracic | **0.70** | **0.98*** | **0.97*** | **0.96*** |  | Lower Thoracic | 0.28* | **0.89*** | **0.89*** | **0.91*** |

| B. TF-NMF analysis | | | | | | | | | | |
| --- | --- | --- | --- | --- | --- | --- | --- | --- | --- | --- |
| Correlation of spatial patterns | | | | |  | **Correlation of temporal patterns** | | | | |
| M1 | ND | Upper Cervical | Lower Cervical | Upper Thoracic |  | **M1** | ND | Upper Cervical | Lower Cervical | Upper Thoracic |
| Upper Cervical | -0.65 |  |  |  |  | Upper Cervical | 0.11 |  |  |  |
| Lower Cervical | -0.79* | **0.81*** |  |  |  | Lower Cervical | 0.30* | **0.97*** |  |  |
| Upper Thoracic | -0.60 | **0.87*** | **0.91*** |  |  | Upper Thoracic | -0.06 | **0.98*** | **0.91*** |  |
| Lower Thoracic | -0.58 | **0.89*** | **0.83*** | **0.90*** |  | Lower Thoracic | -0.08 | **0.94*** | **0.88*** | **0.95*** |
| M2 |  |  |  |  |  | **M2** |  |  |  |  |
| Upper Cervical | -0.64 |  |  |  |  | Upper Cervical | -0.03 |  |  |  |
| Lower Cervical | -0.90* | **0.71** |  |  |  | Lower Cervical | 0.16 | **0.97*** |  |  |
| Upper Thoracic | -0.65 | **0.92*** | **0.79*** |  |  | Upper Thoracic | -0.05 | **0.99*** | **0.95*** |  |
| Lower Thoracic | -0.81* | **0.80*** | **0.86*** | **0.74** |  | Lower Thoracic | 0.14 | **0.94*** | **0.95*** | **0.93*** |
| M3 |  |  |  |  |  | **M3** |  |  |  |  |
| Upper Cervical | 0.15 |  |  |  |  | Upper Cervical | **0.79*** |  |  |  |
| Lower Cervical | 0.27 | **0.96*** |  |  |  | Lower Cervical | **0.85*** | **0.97*** |  |  |
| Upper Thoracic | 0.18 | **1.00*** | **0.97*** |  |  | Upper Thoracic | **0.80*** | **1.00*** | **0.98*** |  |
| Lower Thoracic | 0.03 | **0.92*** | **0.95*** | **0.94*** |  | Lower Thoracic | **0.86*** | **0.93*** | **0.97*** | **0.94*** |

Bold text indicates a strong correlation (r≥0.7). Asterisk indicates statistical significance (*p*<0.05).

**Supplementary Table 4. Correlation of SF- and TF-NMF 3-module patterns among ND and YSI categories**

| A. SF-NMF analysis | | | | | | | | | | | | | | |
| --- | --- | --- | --- | --- | --- | --- | --- | --- | --- | --- | --- | --- | --- | --- |
| Correlation of spatial patterns | | | | | | |  | **Correlation of temporal patterns** | | | | | | |
| M1 | ND | ≤ 1 yr | 1-2 yrs | 2-3 yrs | 3-5 yrs | 5-10 yrs |  | **M1** | ND | ≤ 1 yr | 1-2 yrs | 2-3 yrs | 3-5 yrs | 5-10 yrs |
| ≤ 1 yr | **0.85*** |  |  |  |  |  |  | ≤ 1 yr | 0.52* |  |  |  |  |  |
| 1-2 yrs | **0.90*** | **0.94*** |  |  |  |  |  | 1-2 yrs | 0.19 | **0.88*** |  |  |  |  |
| 2-3 yrs | **0.92*** | **0.96*** | **0.98*** |  |  |  |  | 2-3 yrs | 0.52* | **0.92*** | **0.91*** |  |  |  |
| 3-5 yrs | **0.95*** | **0.86*** | **0.94*** | **0.92*** |  |  |  | 3-5 yrs | 0.55* | **0.72*** | **0.73*** | **0.86*** |  |  |
| 5-10 yrs | **0.82*** | **0.96*** | **0.98*** | **0.98*** | **0.87*** |  |  | 5-10 yrs | 0.15 | **0.89*** | **0.98*** | **0.88*** | 0.65* |  |
| > 10 yrs | **0.83*** | **0.93*** | **0.83*** | **0.92*** | **0.74** | **0.89*** |  | > 10 yrs | 0.27* | **0.93*** | **0.93*** | **0.92*** | **0.71*** | **0.96*** |
| M2 |  |  |  |  |  |  |  | **M2** |  |  |  |  |  |  |
| ≤ 1 yr | **0.83*** |  |  |  |  |  |  | ≤ 1 yr | 0.31* |  |  |  |  |  |
| 1-2 yrs | **0.87*** | **0.96*** |  |  |  |  |  | 1-2 yrs | 0.03 | **0.84*** |  |  |  |  |
| 2-3 yrs | **0.88*** | **0.88*** | **0.90*** |  |  |  |  | 2-3 yrs | 0.34* | 0.63* | **0.85*** |  |  |  |
| 3-5 yrs | **0.89*** | **0.87*** | **0.93*** | **0.89*** |  |  |  | 3-5 yrs | 0.44* | 0.54* | **0.85*** | **0.92*** |  |  |
| 5-10 yrs | **0.79*** | **0.99*** | **0.94*** | **0.90*** | **0.84*** |  |  | 5-10 yrs | -0.04 | **0.90*** | **0.98*** | **0.79*** | **0.74*** |  |
| > 10 yrs | 0.50 | 0.55 | 0.40 | 0.57 | 0.23 | 0.59 |  | > 10 yrs | 0.50* | 0.36* | 0.66* | 0.60* | **0.79*** | 0.59* |
| M3 |  |  |  |  |  |  |  | **M3** |  |  |  |  |  |  |
| ≤ 1 yr | 0.39 |  |  |  |  |  |  | ≤ 1 yr | 0.37* |  |  |  |  |  |
| 1-2 yrs | 0.62 | **0.92*** |  |  |  |  |  | 1-2 yrs | 0.19 | **0.97*** |  |  |  |  |
| 2-3 yrs | 0.65 | **0.91*** | **0.99*** |  |  |  |  | 2-3 yrs | 0.35* | **0.98*** | **0.95*** |  |  |  |
| 3-5 yrs | 0.57 | **0.93*** | **0.99*** | **0.98*** |  |  |  | 3-5 yrs | 0.38* | **0.97*** | **0.93*** | **0.98*** |  |  |
| 5-10 yrs | 0.69 | **0.88*** | **0.99*** | **0.98*** | **0.98*** |  |  | 5-10 yrs | 0.16 | **0.96*** | **0.98*** | **0.97*** | **0.93*** |  |
| > 10 yrs | 0.34 | **0.96*** | **0.92*** | **0.92*** | **0.95*** | **0.87*** |  | > 10 yrs | 0.38* | **0.94*** | **0.88*** | **0.95*** | **0.99*** | **0.89*** |

| B. TF-NMF analysis | | | | | | | | | | | | | | |
| --- | --- | --- | --- | --- | --- | --- | --- | --- | --- | --- | --- | --- | --- | --- |
| Correlation of spatial patterns | | | | | | |  | **Correlation of temporal patterns** | | | | | | |
| M1 | ND | ≤ 1 yr | 1-2 yrs | 2-3 yrs | 3-5 yrs | 5-10 yrs |  | **M1** | ND | ≤ 1 yr | 1-2 yrs | 2-3 yrs | 3-5 yrs | 5-10 yrs |
| ≤ 1 yr | -0.76* |  |  |  |  |  |  | ≤ 1 yr | -0.03 |  |  |  |  |  |
| 1-2 yrs | -0.71 | **0.87*** |  |  |  |  |  | 1-2 yrs | 0.23* | **0.95*** |  |  |  |  |
| 2-3 yrs | -0.68 | **0.83*** | **0.89*** |  |  |  |  | 2-3 yrs | 0.23* | **0.96*** | **0.98*** |  |  |  |
| 3-5 yrs | -0.87* | **0.94*** | **0.92*** | **0.87*** |  |  |  | 3-5 yrs | -0.06 | **0.98*** | **0.92*** | **0.93*** |  |  |
| 5-10 yrs | -0.53 | **0.84*** | **0.82*** | **0.94*** | **0.78*** |  |  | 5-10 yrs | 0.05 | **0.98*** | **0.96*** | **0.97*** | **0.99*** |  |
| > 10 yrs | -0.84* | **0.98*** | **0.87*** | **0.89*** | **0.96*** | **0.87*** |  | > 10 yrs | 0.43* | **0.79*** | **0.87*** | **0.92*** | **0.78*** | **0.85*** |
| M2 |  |  |  |  |  |  |  | **M2** |  |  |  |  |  |  |
| ≤ 1 yr | -0.80* |  |  |  |  |  |  | ≤ 1 yr | 0.13 |  |  |  |  |  |
| 1-2 yrs | -0.66 | 0.60 |  |  |  |  |  | 1-2 yrs | 0.05 | **0.94*** |  |  |  |  |
| 2-3 yrs | -0.78* | 0.60 | **0.91*** |  |  |  |  | 2-3 yrs | 0.07 | **0.96*** | **0.98*** |  |  |  |
| 3-5 yrs | -0.56 | 0.59 | **0.91*** | **0.91*** |  |  |  | 3-5 yrs | -0.10 | **0.90*** | **0.96*** | **0.94*** |  |  |
| 5-10 yrs | -0.72 | 0.48 | **0.94*** | **0.86*** | **0.74** |  |  | 5-10 yrs | 0.14 | **0.94*** | **0.98*** | **0.96*** | **0.96*** |  |
| > 10 yrs | -0.59 | 0.22 | 0.18 | 0.48 | 0.27 | 0.26 |  | > 10 yrs | -0.23* | **0.76*** | **0.80*** | **0.79*** | **0.90*** | **0.78*** |
| M3 |  |  |  |  |  |  |  | **M3** |  |  |  |  |  |  |
| ≤ 1 yr | 0.59 |  |  |  |  |  |  | ≤ 1 yr | **0.90*** |  |  |  |  |  |
| 1-2 yrs | 0.30 | **0.92*** |  |  |  |  |  | 1-2 yrs | **0.81*** | **0.97*** |  |  |  |  |
| 2-3 yrs | 0.23 | **0.87*** | **0.99*** |  |  |  |  | 2-3 yrs | **0.83*** | **0.95*** | **0.97*** |  |  |  |
| 3-5 yrs | 0.17 | **0.86*** | **0.98*** | **0.98*** |  |  |  | 3-5 yrs | **0.78*** | **0.92*** | **0.94*** | **0.98*** |  |  |
| 5-10 yrs | 0.07 | **0.71** | **0.92*** | **0.94*** | **0.95*** |  |  | 5-10 yrs | **0.80*** | **0.94*** | **0.98*** | **0.96*** | **0.97*** |  |
| > 10 yrs | 0.18 | **0.82*** | **0.83*** | **0.83*** | **0.86*** | **0.69** |  | > 10 yrs | 0.68* | **0.81*** | **0.84*** | **0.92*** | **0.96*** | **0.87*** |

Bold text indicates a strong correlation (r ≥ 0.7). Asterisk indicates statistical significance (*p*<0.05).
